# Modeling and empirical evidence of the impact of implementation of sugar sweetened-beverages tax to reduce non-communicable diseases prevalence: a systematic review

**DOI:** 10.3389/fnut.2024.1448300

**Published:** 2024-10-11

**Authors:** Safira Firdaus, Nuri Andarwulan, Purwiyatno Hariyadi

**Affiliations:** ^1^Department of Food Science and Technology, IPB University, Bogor, Indonesia; ^2^South-East Asia Food and Agriculture Science and Technology (SEAFAST) Center, IPB University, Bogor, Indonesia

**Keywords:** evaluation, evidence, NCD, SSB, simulation, tax

## Abstract

**Introduction:**

The surge in non-communicable diseases (NCDs) has been linked to excessive sugar-sweetened beverage (SSB) consumption. In response, the World Health Organization advocates for SSB taxes as a preventive measure. This study conducts a systematic literature review, encompassing simulation modeling and empirical evidence, to evaluate the effectiveness of SSB taxes in diminishing NCD prevalence.

**Method:**

A systematic search from August 2002 to August 2022, utilizing databases like ScienceDirect, PubMed, Google Scholar, Wiley Online Library, Springer, and ResearchGate, identified 29 relevant articles worldwide following PRISMA 2020. The Critical Appraisal Skill Programme (CASP) 2018 tool was employed for economic evaluation

**Result:**

Among the selected articles, 22 utilized simulation models in group of countries such as South Africa, the US, the UK, Asia (Philippines, India, Indonesia, Thailand), Australia, and Europe (Germany), while seven were based on US and UK evidence. Simulation modeling consistently demonstrated that SSB taxes significantly reduced NCD incidence, prevalence, and mortality, also bolstering government revenue. Tax rates in simulations ranged from 10 to 25%. However, empirical evidence indicated a limited impact, primarily due to low tax rates. Notably, a UK-specific tax led to a 2.7% reduction in SSB sugar purchases and 40.2% higher purchases of no-levy drinks.

**Discussion:**

The findings suggest that while simulation models demonstrate the potential effectiveness of SSB taxes in reducing NCDs, empirical evidence reveals there is no significant effect of the SSB tax, Based on the study conducted in this study, the SSB tax is not effective in reducing the prevalence of NCDs due to consumer preferences that have not changed. , likely due to the implementation of lower tax rates and failure to fulfill the assumption of subtitution product, physical activity, and so on. The study highlights that SSB tax is not effective in reducing the prevalence of NCDs due to consumer preference that have not change. Multi-actions are needed to support the sustainability of the implementation of the SSB tax, including education and promotion of healthy lifestyles and encouragement to reformulate SSB products by industry.

## 1 Introduction

Sugar-sweetened beverages (SSB) or sweetened drinks are beverages that contain ‘free sugar’ (1) or ‘added sugar’ in their production, which can be added during cooking, and serving, like High-Fructose-Corn-Syrup (HFCS) or naturally occurring in honey, fruit concentrates, syrups, and fruit juices (1–3). SSB includes carbonated and non-carbonated drinks, fruit juices, ready-to-drink coffee, ready-to-drink tea, fruit-flavored beverages, flavored milk drinks, energy drinks, and vitamin water (4, 5). SSB contributes to at least 10–15% of calorie intake in children (6). In Jordan, calories consumed from the SSB was 480.6 ± 338.89 kcal/day among 1,000 students aged between 18 and 25 years (7). The WHO recommends that sugar intake should not exceed 10% of the total energy intake for both children and adults and even recommends reducing sugar consumption to below 5% under specific conditions (8).

The high consumption of SSB has led to adverse health impacts, such as an 18% increase in the risk of type 2 diabetes mellitus (T2DM) per day (9, 10), which T2DM is one of the top 10 diseases that increase the burden globally, where diabetes contributed the largest number of DALYs in the last 30 years at 24.4% according to the age-standardized DALY rate (11). SSB consumption is also associated with weight gain, obesity, overweight, and dental problems in children (decayed-missing-filled teeth) (12); the risk of cardiovascular diseases (CVD), more than 100,000 men and women were analyzed, it was found that a person who consumed two or more servings per day had a 31% higher risk of death from CVD compared to a person who consumed less than one serving per month (13). This risk may be caused by high coronary artery calcium, triglycerides, C-reactive protein, pulse wave velocity, etc. For example, a study of more than 22,000 men and women of median age of 40 years, reported significantly higher coronary artery calcium scores in the highest SSB-consuming cohort (more than five SSB drinks/week) versus non-SSB drinkers (CAC ratio: 1.70; 95% CI 1.03, 2.81). High coronary artery calcium relates to the potential risk of coronary artery occlusion and myocardial infarction which may cause death (14). Higher SSB intake also causes cancer due to being overweight (15). Overweight is a strong risk factor for mouth, pharynx, larynx, stomach, pancreatic, liver, gallbladder, colorectal, breast, endometrial, ovarian, prostate, and kidney cancers. SSB might promote visceral adiposity of body weight, which might promote tumorigenesis through alteration in cell signaling pathways and adipokine secretion and rapidly absorbed carbohydrates were associated with breast carcinogenesis (15).

Based on statistical data, North America ranks first as the country with the highest consumption of packaged sugary drinks in the world (2013–2017) with carbonated beverage consumption (±110 L *per capita*) in first place followed by fruit juices, ready-to-drink products, and energy drinks, while South Asia ranks last (±10 L *per capita*). This consumption amount is expected to continue to increase in 2022 for countries in Latin America, the Caribbean, Europe, Central Asia, the Middle East, North Africa, East Asia and the Pacific, and South Asia (16). Hashem et al. (17) in their research on the amount of free sugar and calories contained in carbonated sweetened beverages stated that the average free sugar content in a 330 ml package is 30.1 ± 10.7 grams. Globally, the diet’s largest source of added sugar is SSBs; a 12 fl oz. (355 ml) serving of soda contains 35.0–37.5 g of sugar and 140–150 calories (10).

The increasing prevalence and mortality rates of non-communicable diseases (NCDs) associated with SSB have prompted various parties to address this complex issue, starting with changing sugar consumption patterns to reduce obesity and overweight. WHO recommends in their ‘Best Buy’ that an effective way to reduce sugar intake is by implementing an SSB tax (3, 5). One of the goals of implementing an SSB tax was to combat obesity (16). In addition to health benefits, the SSB tax provides substantial economic advantages that can be used to enhance healthcare facilities and public health equalization programs (18, 19).

Numerous studies have been conducted on the impact of SSB taxation in reducing the prevalence of NCDs, both through estimation via modeling and based on evidence after the implementation of SSB taxation. Previous studies have reported scoping reviews related to modeling the implementation of SSB taxes (20), and a meta-analysis of evidence has shown that SSB taxes reduce obesity prevalence (21). To understand the progress, effectiveness, and advanced research on SSB taxation based on modeling and empirical evidence, it was essential to synthesize existing studies, identify their strengths and weaknesses, and present more comprehensive data. Therefore, we conducted a systematic literature review to describe and compare the impact of SSB taxation in reducing the prevalence of NCD based on the modeling literature and empirical evidence (see Supplementary Table 1).

The goal of this review is to gather comprehensive information that can be used as a source for appropriate policy development and a reference for research gaps. The questions we aim to answer are as follows:(1) How does the implementation of SSB taxation, based on modeling and empirical evidence, affect the prevalence of NCDs? (2) Is SSB taxation the most effective way to reduce NCD prevalence globally, based on both modeling and empirical evidence?

## 2 Methods

We used a systematic literature review as our method that was performed by the PRISMA 2020. We used the Population-Intervention-Control-Outcome (PICO) approach to formulate the research question: “Is there a difference in the prevalence of NCD in humans resulting from the implementation of SSB taxation based on modeling compared to empirical evidence?” (22, 23). We conducted a systematic search from August 2002 to August 2022. The following databases were searched: ScienceDirect, PubMed, Google Scholar, Wiley Online Library, Springer, and ResearchGate, using relevant keywords such as “sugar,” “sugary,” “foods” OR “food,” “health,” “tax,” “taxation,” “sweet,” “benefit,” “advantage,” “elasticity AND nutrition,” “effective,” “obesity,” “diabetes,” “cardiovascular,” “cancer,” “prevalence,” “evidence,” “incident,” “case,” “simulation,” “beverage,” and “modeling.”

Studies eligible for inclusion in this systematic review are: (i) studies that demonstrated and explained the impact of SSB tax on reducing the prevalence of NCD, including simulation modeling and evidence-based; (ii) studies that the subjects were men and women of all ages; (iii) open access and full text; (iv) written in English; (v) published between August 2002 and August 2022. This review excludes any other systematic review and meta-analysis, case studies, editorial, and reports. We also exclude the studies that explain taxation on sugary products in another form than SSB.

We used a free reference manager (Mendeley Desktop 1.19.8) for duplicate documents, sorting, and search-time optimization. The selected articles were extracted, and sorted by Ms. Excel 2019. The required data include general article information (authors, year of publication, country, population/sample), fiscal parameters (see Supplementary Table 2), and health outcomes (BMI, overweight and obesity prevalence, T2DM, CVD, cancer, stroke, dental carries, quality of life, and mortality rates). Each eligible article was assessed using the Critical Appraisal Skill Programme for economic evaluation (24). We applied the above quality criteria for all eligible studies and rated each of them on 12 criteria, establishing the presence of ‘1 point’ or the absence of ‘0 point’ for each item. All studies that achieved a score equal to or higher than 6 points in these quality criteria were selected for this systematic review report.

## 3 Result and discussion

**Figure 1:**
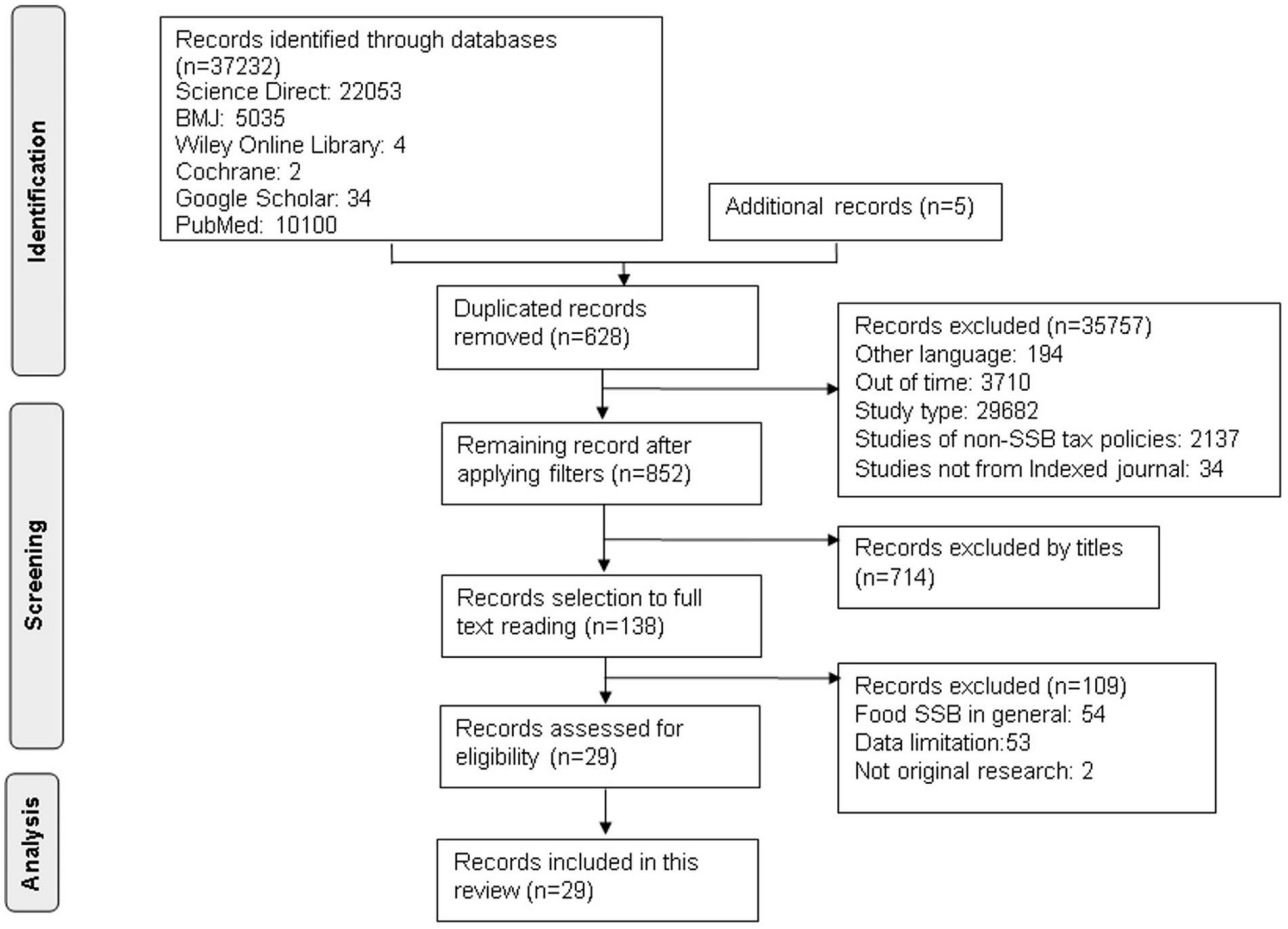
Flow chart of the selection of studies evaluating the impact of SSB tax on NCDs.

Sugar-sweetened beverages have become increasingly popular in recent years, contributing to rising rates of obesity and related health problems. To better understand the impact of sugar-sweetened beverage consumption on obesity and other adverse health outcomes, researchers have utilized simulation models and examined the available evidence (25). Figure 1 shows the results of the literature search. A total of 37,232 articles were identified after the database search as well as five additional records from the reference list. A total of 628 duplicate articles were identified; therefore, deduplication was performed using Mendeley. A total of 35,757 articles were excluded during the screening stage because they were in other language than English, exceeded the publication time in the inclusion criteria, were not related to SSB, and were not published in an indexed journal. Of the 852 articles, 714 did not meet the inclusion criteria, using filtering features in each database based on the alignment of titles and abstracts with predefined keywords. Subsequently, further selection was performed based on the exclusion criteria, resulting in articles falling under the exclusion criteria, leaving 138 complete articles that could be utilized. After re-reading, 54 articles were excluded for discussing taxes on all food product categories, 53 articles were excluded for not providing information on tax design, and two other articles utilized the same data. A total of 29 articles were used for data analysis and synthesis. These articles were sourced from South Africa, America (Brazil, the USA, Canada, and Mexico), Australia, Asia (India, Indonesia, Philippines, Thailand), and the United Kingdom (Ireland and England). All the articles were published between 2006 and 2022. Subsequently, a study quality assessment was conducted based on the questions listed in Supplementary Table 3.

A total of 22 articles included tax application modeling using a comparative risk assessment Modeling study (7), Mathematical Simulation (1), Cohort Study (1), microsimulation (4), Proportional Multi-state Life Table (4), Cost-effectiveness Analysis (3), econometric and epidemiological (1), and System Dynamics Model (1) (see Supplementary Table 4). The remaining seven articles were tax application articles with cross-sectional, repeated cross-sectional, longitudinal, and longitudinal comparative case studies and controlled interrupted time-series analysis.

### 3.1 The impact of implementing SSB taxation on NCDs based on simulation models

Based on these findings, 22 selected articles encompassing tax implementation modeling from around the world, such as South Africa, America (Brazil, the USA, Canada, and Mexico), Australia, Asia (India, Indonesia, Philippines, Thailand), and the United Kingdom (Ireland and England), reported findings regarding the reduction in the prevalence of obesity, overweight, BMI, diabetes, CVD, stroke, ischemic heart disease (IHD), Disabilty-Adjusted-Life-Years (DALYs), Quality-Adjusted-Life-Years (QALYs), Health-Adjusted-Life-Years (HALYs), and dental caries.

Significant results were demonstrated in 21 articles (95%), indicating that SSB taxes were effective in reducing the prevalence of NCDs (see Supplementary Table 5). The study population tended to belong to an adult age group. The population used in each study was based on the age group’s tendency to consume SSB in a particular country. Findings utilizing adult populations include those reported by Fletcher et al. (26) and Lin et al. (27), Briggs et al. (28), Briggs et al. (29), Basu et al. (30), Veerman et al. (31), Barrientos-Gutierrez et al. (32), Bourke and Veerman (33), Saxena et al. (34), Saxena et al. (35), Sowa et al. (36), Wilde et al. (19), with the age range of the studied population being above 16–85 years old.

To develop simulation models, each researcher utilized a specific simulation design. The simulated tax rates varied between 1 and 25% in each model, with the most frequently used rates between 10 and 20%. The modeling designs used included Comparative Risk Assessment Modeling Studies or CRAM (27, 28, 37–40); mathematical simulations (41), cohort study (36), and microsimulations (19, 30, 32, 42), the Proportional Multi-state Life-Table model (PMSLT) (31, 33, 43, 44), Cost-effectiveness Analysis or CEA (18, 34, 35), Econometric-Epidemiologic (45), and System Dynamics Model (46). The simulation modeling design methods are detailed in Supplementary Table 6, and the population and results are detailed in Supplementary Table 7.

Based on the findings of the simulation model, we found six articles (26, 28, 29, 37, 41, 45) that did not clearly state the time horizon used in their research simulation articles explain the assumptions used in carrying out the simulation and carry out uncertainty, validity, and sensitivity analysis to check the robustness of their results. The types of tax rates used in the articles we reviewed were diverse, namely ad valorem, volumetric, and specific tax. Almost all of the simulation-based articles that we reviewed used own-price elasticity values to estimate consumer responses to purchasing SSB products after the tax was applied. The average value of own-price elasticities used was −1.29 (21). However, only a few studies use cross-price elasticity values to estimate consumer responses to purchasing substitute products such as milk, mineral water, juice, coffee, and tea [ex. (27, 28)].

#### 3.1.1 The SSB tax impact estimation on BMI

Various health parameters such as BMI, obesity status, overweight status, CVD status, and dental caries status were used to estimate the effectiveness of the tax. The prevalence of NCDs was determined by conducting simulations of health parameters to obtain results will be obtained on the prevalence of NCDs, quality of life, and economic benefits. Based on the results of the alleged effectiveness of SSB tax on BMI, four studies have shown the significance of reducing BMI, Lin et al. (27), Long et al. (18), Barrientos-Gutierrez et al. (32) and Liu et al. (44).

However, the SSB tax, which has a low rate, does not significantly reduce BMI (−0.003%; 0.0004 < 0.01) (38). This was thought to be due to the low rates used in the modeling simulation; therefore, it does not have a real impact on the population. The average decrease in BMI is 0.15 Kg/m^2^ ± 0.55 for a 10% tax rate with a monthly peak reach of 0.29% ± 0.01, and doubles for a 20% tax rate (32, 44). The SSB tax impact estimation for the BMI is shown in Supplementary Table 5.

#### 3.1.2 The SSB tax impact estimation on obesity

The alleged effectiveness of SSB tax on the prevalence of obesity has been simulated by various models including mathematical simulation (41), microsimulation (30, 32, 42), and PMSLT (31, 33, 43), cost-effectiveness (18), and CRAM (26–29, 37, 40), and econometric-epidemiologic (45). Each simulation model showed varying levels of significance in reducing obesity prevalence (Supplementary Table 5). The use of a 10% tax rate was estimated to reduce the prevalence of obesity by 1.3–2.4%, while the use of a 20% tax rate reduces at least 400,000 new cases of obesity (43), and reach 11.75% within 10 years.

Briggs et al. (37) used a specific tax. In 2017, it was proven to be more effective in reducing the prevalence of obesity because it reduced at least 81,594 new cases per year (3,588–182,669; 0.5%). This was because of the appropriate tax rate design, namely, a specific tax will affect the SSB industry, where SSB with a sugar content exceeding 8 g/100 ml will be subject to a rate of £24 penny/L, while drinks containing 5–8 g/100 ml sugar will be subject to rate £18penny/L. (47).

#### 3.1.3 The SSB tax impact estimation on overweight

The BMI parameters obtained from a population were also used to determine overweight and obesity status, which were affected by the implementation of the SSB tax. Supplementary Table 5 shows two simulation models that measure the prevalence of overweight: PMSLT and CRAM. Both models showed a significant reduction in case prevalence ranging from 0.9–3% using rates of 10–20% (27–29, 33, 40, 43, 44). However, the prevalence of overweight did not decrease significantly (−0.0002%; 0.0000 > 0.01) when the tax rate was 1% (38). It was suspected that low tax rates and not considering substituting products when conducting simulations could cause insignificant results (21, 41, 48).

#### 3.1.4 The SSB tax impact estimation on T2DM

Type 2 diabetes mellitus (T2DM) is an NCD that is most at risk when consuming excess SSB. Based on the studies analyzed, the prevalence of T2DM has decreased significantly, and the application of specific rates can reduce 10,681 (3,899–18,964; 17,7) new T2DM cases each year (37). These results were higher than those obtained using the PMSLT simulation model with a 20% tax rate, where there was a reduction of 60,000–145,000 cases within 25 years of the tax implementation (31, 33, 43). This lower estimate was caused by using ad valorem tax (percentage rate) because specific rates affect the SSB industry. The specific tax causes sugar reformulation, and estimates of reducing the prevalence of T2DM were targeted (1, 3).

In Indonesia, the incidence of T2DM that can be estimated due to the implementation of tax shows a better reduction impact in the high-income group (Q5), with 1,487,000 cases (95% CI: −3,068,603 to −545,913), or approximately 8.8% (33). This was because the SSB consumption level of the high-income groups was higher than that of the low-income groups (Q1). However, the SSB tax can also affect the lower economic group (Q1) in Canada in 32,600 cases (43). This difference is caused by the different SSB consumption patterns in each region or country as well as the affordability of SSB products in terms of price and ease of obtaining (33, 43). SSB was often consumed in Canada by all groups, especially low-income groups. However, in Indonesia, SSB products were more difficult for low-income groups to obtain SSB products. The SSB tax impact estimations for T2DM incidence and prevalence are shown in Supplementary Table 5.

#### 3.1.5 The SSB tax impact estimation on CVD

Heart attacks or myocardial infarction (MI) and ischemic heart disease can also be estimated to reduce cases through microsimulation modeling, PMSLT, and CEA (Supplementary Table 5). These findings suggest a significant reduction in CVD (19, 31, 33, 34, 43, 44). It was also found that income influenced the estimated reduction in CVD cases using the PMSLT simulation model.

In Indonesia, SSB taxes affect high-income groups more as seen in the 2.1% (−48,168; 95% CI: −58,765 to −38,724) reduction in CVD (33). This was inversely proportional to the research conducted by Kao et al. (43) in Canada, where the low-income group (Q1) was significantly affected by the implementation of taxes on SSB, amounting to 9,632 cases over 25 years. As explained previously, the SSB consumption pattern in Indonesia was in the high-income group, whereas in Canada, the low-income group consumes more SSB than the high-income group.

#### 3.1.6 The SSB tax impact estimation on dental caries

The SSB tax also affects dental health, including dental caries. Higher sugar intake from SSB can cause tooth decay and loss (decayed-missing-filled teeth/DMFT) (36). Based on existing findings, the SSB tax is effective in reducing cases and the prevalence of dental caries Supplementary Table 5. However, based on system dynamics model simulations, the results obtained have a small magnitude (1%), where SSB tax affects groups with low levels of dental caries severity more than those with severe levels of dental caries (46). Estimating the reduction in the prevalence of dental caries using a system dynamics model shows a complex relationship between tax, sugar consumption, and oral health, which was supported by dental health facilities and the behavior of consumers themselves (46).

According to Briggs et al. (37), a decrease in the prevalence of dental caries can occur as soon as possible after the SSB tax is implemented, so the results of the reduction need to be considered when applying specific tax rates. The sudden shift in people’s consumption patterns from drinks with high sugar content to drinks with low sugar content has a temporal impact, which can cause bias in the estimation results using modeling simulations. In their research, Briggs et al. (37) also stated that if there was a scenario of reformulating the sugar content in SSB combined with the implementation of the tax, it would further accelerate the reduction in the prevalence of dental caries by 269,375 cases (82,211–470,928; 4.4% per 1,000 people per year).

#### 3.1.7 The SSB tax impact estimation on cancer

Supplementary Table 5 shows the SSB tax impact estimation for cancer incidents. The Canadian population was simulated by Kao et al. (43) and Liu et al. (44) using the PMSLT model, but the rates applied were different, namely ad valorem tax (20%) and volumetric tax (CAD$0.015/oz). The simulated population was 20 years of age. Based on these findings, the SSB tax was effective in reducing cases and the prevalence of cancer. Overall, the incidence of new cancers decreased by 17,740 cases, with a decrease in the prevalence of breast cancer cases by 1.2% (Q1) and 0.9% (Q5), thyroid cancer by 0.7% (Q1) and 0.6% (Q5), and colon and rectum cancer by 0.5% (Q1) and 0.4% (Q5) per 100,000 cases (43). The SSB tax influences the decline in cancer cases in Q1 (quintile 1), *low income*, which was in line with the simulations of Schwendicke and Stolpe (40) and Wilde et al. (19). Meanwhile, the application of a volumetric tax showed that at least 1,451 (95% CI: 1,186–1,708) new cases of breast cancer, 522 (95% CI:477–567) cases of thyroid cancer, and 233 (95% CI: 223–243) cases of colon and rectal cancer were prevented (44). The current epidemiological evidence shows that sugary drink consumption is linked with breast cancer, of the 927 breast cancer cases, 386 patients (54.7%) died by the end of the follow-up period. Compared with those who never/rarely consumed SSBs, consumption more than 5 times per week was associated with an increased risk of death from total cancer (95% CI: 1.16–2.26; HR: 1.62; *P*trend < 0.01) and breast cancer (95% CI:1.16–2.94; HR: 1.85; *P*trend < 0.01) (49). Besides breast cancer, colorectal cancer risk also increased when the consumption of sugary drinks increased, of the 101,257 participants, 166 new colorectal cancer incidents were found (95% CI:0.84–1.46; HR: 1.85; *P*trend: 0.50) (15).

The investigation included 450,064 adults from the European Prospective Investigation into Cancer and Nutrition (EPIC) cohort, which shows that after 14 years a dietary pattern rich in sweetened beverages was positively associated with differentiated thyroid cancer risk (95% CI:0.99–1.61; HR_Q4vs.Q1_: 1.26; *P*trend: 0.07). The intake of sweetened beverages was positively associated with differentiated TC risk (95% CI:1.00–1.11; HR_100ml/d_: 1.05), especially with papillary thyroid risk (95% CI:1.01–1.13; HR_100ml/d_: 1.07). Similar results were observed with sugary and artificially sweetened beverages (50).

#### 3.1.8 The SSB tax impact estimation on stroke

Estimates for reducing the incidence and prevalence of stroke were simulated using three types of models: microsimulation (19), PMSLT (31, 33, 43), and CEA (43) (see Supplementary Table 5). Based on a microsimulation with a rate of $ 0.01/ounce, it was successful in estimating a decrease in stroke cases (49) of 60 (95% CI:81 to 181) cases/million adults, where the SSB tax had a greater impact on groups of people who did not have health insurance, experiencing a decrease in stroke cases of −379 (95% CI:-619 to −203) cases/million adults (19). This was because of the high consumption of SSB in groups that do not have health insurance. Based on a cost-effectiveness simulation with a rate of 13%, stroke cases have decreased by 19,858 cases in the Philippines, where the SSB tax has a greater impact on the Q5 or high-income group by 5,139 cases (34). This statement was supported by estimates in Indonesia, with a price increase of 20%, and the prevalence of stroke decreased significantly in Q5 (2.1%; −44,746 cases; 95% CI: −56,744 to −34,291) (33). It was suspected that SSB consumption in Q5 was higher in the Philippines and Indonesia than in countries in America and Europe; therefore, the tax affects quintile 5 more than quintile 1.

#### 3.1.9 The SSB tax impact estimation on mortality

Death cases were related to diseases caused by excessive SSB consumption (see Supplementary Table 8). With the implementation of the SSB tax, deaths associated with ischemic heart disease, T2DM, and stroke can be estimated using four models: microsimulation (19), and PMSLT (31, 44), CEA (34, 35), and CRAM (39). By simulating a 10% rate in southern Africa, at least 8,000 deaths due to SSB consumption can be prevented (35). The 13% tax rate simulated in the Philippines, overall prevents deaths due to T2DM 5,913 cases, IHD 10,339 cases, and stroke 7,950 cases, where SSB tax has more influence on preventing deaths in quintile 1, low income, than quintile 5. This was due to low SSB consumption in quintile group 1 (34).

A simulated tax rate of 20% shows a reduction in deaths of 13.7% or 5,386 (95% UI: 5,074–5,727) for males and 12.7% or 6,075 (95% UI: 5,649–6,531) for females in Brazil within 10 years (39) and prevents 1,600 cases associated with IHD in Australia within 25 years. Years (31). This result was not much different from microsimulation in America, where the rate used was a volumetric tax ($0.01/ounce), where preventable deaths due to IHD were 1,540 (95% CI: 995 to 2,118) cases/million adult people (19). In Canada, by simulating a tax rate of CAD$0.015/oz., 2,189 (95% CI: 1,866 to 2,447) deaths could be prevented over 25 years (44).

#### 3.1.10 The SSB tax impact estimation on daily calorie intake

Daily calorie intake has a significant impact, based on estimates obtained through mathematical simulation modeling (41), PMSLT (33), CEA (18), CRAM (28, 29) and econometric-epidemiologic (45) (see Supplementary Table 8). By simulating a 10% SSB tax rate, in America, daily calorie intake will experience a reduction of 1.56 kcal/day for the age group 2–19 years, but daily calorie intake will experience a smaller reduction for the age group over 19 years (0.90 kcal/ day) (18). Meanwhile, in Ireland, a 10% tax rate was able to reduce 2.1 kcal/day for the age group over 19 years, where men experienced a higher reduction in daily calorie intake than women (29).

With a 20% price increase, there was a calorie reduction of 16.7 kJ/people/day in the UK (28), equivalent to estimates in Indonesia where the calorie reduction was 17 kJ/people/day (33), and almost a halving. Times higher than that in South Africa by as much as 36 kJ/day (41). However, the estimation results carried out in Thailand experienced a spike in estimates, namely 109.6 kJ/people/day (45). In line with estimates made by Briggs et al. (28), daily calorie reduction affects men (367 kJ/capita) more than women with low income (40). Apart from the 10 and 20% tax rates, 11 and 25% tax rates were also simulated in Thailand, because in 2021, SSB prices in Thailand experienced a jump of 11%, while the 25% tax rate was chosen based on recommendations from the WHO. The results obtained were approximately 59 kJ/people/day of reduced calories for the 11% tax rate simulation and 134.9 kJ/people/day of reduced calories for the 25% tax rate simulation.

#### 3.1.11 The SSB tax impact estimation on quality of life

The price increase caused by the SSB tax has a significant impact on Quality of Life or QoL (see Supplementary Table 8). The simulation of a tax rate of $0.01/ounce or the equivalent of 10% in America shows an estimated number of disability-adjusted life years (DALYs) that can be prevented by 101,000 (95% UI: 34,800, 249,000), and the number of quality-adjusted life years (QALYs) was 871,000 (95% UI: 342,000, 2,030,000) using the CEA model (18). The number of QALYs that can be estimated through the microsimulation model was four times higher, 3.4 million (95% UI: 1.85, 4.77) in a lifetime (19). Simulations in Canada show that both the ad valorem rate (20%) and volumetric tax (CAD$0.015/oz) affect the DALYs and QALYs. The number of DALYs that can be averted by simulating a 20% rate through the PMSLT model was 760,000 over a lifetime period among the 2016 adult population, with more DALYs averted than females (43), while volumetric tax shows higher results than ad valorem tax, namely 2.29 million DALYs averted and 1.5 million QALYs gained (44). In Australia, the HALYs gained were higher in men than in women (112,000, 95% UI: 73,000–155,000) (31). In line with this, in Indonesia, HALYs affect men more than women, although not too differently (29%) (33).

#### 3.1.12 The SSB tax impact estimation on economic benefit

The implementation of tax not only provides benefits to the health sector but also the economic sector. The benefits can be in the form of health costs, tax revenue, and poverty prevention due to NCDs. Supplementary Table 8 shows that five models were used to estimate the effectiveness of tax on the economic sector: microsimulation (19), cohort model (36), and PMSLT (31, 33, 43, 44), CEA (18, 34, 35), and CRAM (27, 29). The results show that the implementation of SSB tax can increase revenue from taxes. With a simulated rate of 20%, revenue from SSB taxes in the UK was £276 (95% CI: £272 m to £279 m) million/year (28), as well as US$450 million in South Africa with benefits from health costs of US$140 million (35). Simulations using a 20% tax rate were widely used in various countries, such as Australia, Canada, Indonesia, and the US. Especially in Indonesia, US $15.1 (95% CI: 13,703 to 17,295) billion/year (Q5) and US 536 (95% CI: 488 to 607) million/year (Q1) revenue was gained using the PMSLT simulation model (33). In addition, the PMSLT model was also able to estimate the benefits derived from health costs of $37,548 (95% UI: CAD$34,155, 39,784 million) million in Canada (44). Through a CEA simulation model, the reduction in poverty rates could also be estimated as a result of the minimum health costs incurred for handling NCDs in 12,719 cases (35).

### 3.2 The impact of implementing SSB taxation on NCDs based on evidence

Seven articles were found related to the evidence-based decrease in the prevalence of NCDs after the implementation of a tax on SSB and a reduction in SSB purchasing. The research was conducted in the United States and the United Kingdom. Research conducted in the United States has an average tax rate of 1–5%, while research conducted in the UK uses specific tax rates. The Analysis methods and populations are shown in Supplementary Tables 9, 10. Research categories were diverse, including cross-sectional (51), repeated cross-sectional (26, 52), longitudinal (53, 54), longitudinal comparative case studies (48), and controlled time series analysis (55). Based on the evidence, the effectiveness of tax has been studied in reducing BMI, prevalence of overweight and obesity, HOMA-IR resistance values, and its impact on purchase volume and sugar content in SSB. The impact of implementing SSB is shown in Supplementary Table 11.

The results of the SSB tax effectiveness were based on the evidence we reviewed, including time horizon data, as well as the parameters that will be used. In addition, five articles used ordinary least squares regression to calculate the effectiveness of SSB tax on changes in BMI and prevalence of overweight (26, 48, 52–54). The studies state that the tax rates applied in America were in the form of an ad valorem tax. None of the articles included the own-price and cross-price elasticity values used to calculate SSB tax impact. This was caused by limitations on own-price data as well as data on the consumption of substitute products in America when the tax was applied (26, 48).

The increase in prices due to the SSB tax in America has been proven to have no impact on reducing BMI based on research conducted by Powell et al. (52), Fletcher et al. (26), Sturm et al. (54), and Fletcher et al. (48) (see Table 1). Powell et al. (52) stated that consumption of SSB in the teenage age group in America was influenced by the presence of vending machines in schools and residences, however, when the 4.25% (SD: 2.47%; range: 0–7%) tax began to be implemented, the decline in BMI was not significant, with an average of 22.13 Kg/m^2^ or a decrease of around −0.006% (*p value* = 0.09) over 10 years implementation of the tax. Powell et al., observed the purchase of SSB through vending machines and minimarkets because the largest number of SSB purchases according to the survey that had been conducted were located at vending machines, so the ease of purchasing SSB meant that SSB tax had no impact. This was supported by research conducted by Sturm et al. (54), who also conducted research based on rates in the State of 4.2%. The decline in BMI for children after implementing the tax was only around −0.013% (*p value* = 0.10) for every 1% increase in tax rates; therefore, if the tax applied in the state had an average of 4.2%, there would have been a decrease of −0.085% (*p value* = 0.05).

**Table 1:** The SSB tax impact on NCDs based on evidence.

Reference	Design study	Time horizon	Product	Tax rate	Result
(52)	Repeated cross-sectional	10 years	Soft drinks	4.25%	No significant impact on BMI -0.006% (*p value* = 0.09)
(53)	Longitudinal study	20 years	Soda	USD 1/2 L bottle ≈ 37%	−1.05 (95% CI: −1.80, −0.31) Kg/ m^2^ reduction
(26)	Repeated cross-sectional	17 years	Soft drinks	2.27%	No significant impact on BMI −0.015 (*Z score* = 0.016) Kg/m^2^
(54)	Longitudinal study	6 years	Carbonated SSB	4.2%	No significant impact on BMI −0.085% Kg/m^2^(*p value* = 0.05)
(48)	Longitudinal Comparative Case Study	17 years	Soda	2.59%	No significant impact on BMI −0.007 (0.093) Kg/m^2^ (*p value* = 0.937)
**Obesity**					
(51)	Cross-sectional	1991 and 1998	Soft drinks	5%	No significant impact on obesity −1%
(26)	Repeated cross-sectional	17 years	Soft drinks	2.27%	No significant impact on obesity −0.009% (0.006)
**Overweight**					
(26)	Repeated cross-sectional	17 years	Soft drinks	2.27%	No significant impact on overweight −0.002% (0.0011)
**HOMA-IR**					
(53)	Longitudinal study	20 years	Soda	USD 1/ 2 L bottle ≈ 37%	HOMA-IR 0.42 ≤ 1, (95% CI: −0.59, −0.31)

BMI, body mass index; HOMA-IR, homeostatic model assessment for insulin resistance.

Duffey et al. (53) reported that there was a significant change in reducing BMI -1.05 Kg/m^2^ (95% CI: −1.80, −0.31). These results were not much different from the simulations carried out by Lin et al. (27), where the price increase due to tax has an impact on reducing BMI by −0.97 Kg/m^2^ so that both based on evidence and simulation, this was considered very realistic given the environmental conditions for implementing a tax on sweetened drinks in American states.

Fletcher et al. (26, 48) conducted observations on two different types of products, drinks, and soda, where the average tax used in the state was 2.27–2.59%. Fletcher et al. (26) conducted an observational study with a low tax rate (2.27% ± 0.029), which was implemented in around 53 states based on 1996–2006 NHANES data, showing that there was no significant impact on reducing BMI (−0.015 (*Z score* = 0.016) Kg/m^2^), overweight (−0.002%; 0.011), and obesity (−0.009%; 0.006) in adolescents and children (see Supplementary Table 11). These results contradict the simulations carried out by Kristensen et al. (42), and Manyema et al. (41); the SSB tax should affect teenagers and children more because the amount of SSB consumption in this age population is very high due to the ease of obtaining SSB in public places such as schools. However, there has been a shift in consumption patterns for substitute products, with every 1% increase in SSB tax rate increasing calorie intake from whole milk by eight calories per day or around 13% of the average calories from pure milk and increasing the amount of consumption by 11 g. A shift in consumption patterns and an increase in the number of calories from whole milk was a positive response to the increase in SSB tax even though there was no significant reduction in BMI, overweight, and obesity. The high number of calories from whole milk consumed indicates that the SSB tax applied to children and adolescents was ineffective (38).

In 2014, Fletcher et al. (48) tested the non-linear impacts that may arise on the amount of consumption and changes in body weight due to the implementation of the tax on SSB. Non-linear impacts mean substitution impacts that influence the amount of consumption and changes in body weight indirectly when a tax is implemented (48). A substitution impacts analysis was carried out; for every 1% increase in tax, the number of calories from substitute products would increase by 7.5 (3.703; *p value* = 0.0515) calories. The change in BMI after the implementation of the tax was only 0.007 (0.093; *p value* = 0.937) Kg/m^2^, which does not indicate the statistical significance of the implementation of the tax in reducing the number of calories and BMI. This description shows that the results obtained by Fletcher et al. (26) can be refuted where substitute products do not affect the increase in BMI, prevalence of obesity, and overweight as expected.

The increase in prices due to taxes also affected HOMA-IR, a measure of insulin resistance. Duffey et al. (53) conducted a study over 20 years, with a USD 1 per 2 L tax, equal to a 37% price hike in adolescents and adults. HOMA-IR values below 1 indicate higher insulin sensitivity, lowering the risk of Type 2 Diabetes (T2DM) (56). The study found a HOMA-IR value of 0.42 < 1 (95% CI: −0.59, −0.31), suggesting that the volumetric SSB tax in America can lower the risk of T2DM.

The tax also influenced the volume and sugar of SSB purchases. Rogers et al. (55) studied a specific tax’s impact on adults, noting reduced sugar intake despite stable purchasing volumes. The tax rates were GBP 0.24/L for drinks with sugar content ≥8 g/100 ml, GBP 0.18/L for drinks with sugar content ≥5 to <8 g/100 ml, and no tax for drinks with sugar content <5 g/100 ml (55). After the tax, the purchasing pattern of high-tier drinks decreased by 37.8% per household per week and sugar consumption from SSB decreased by 16.2 g. SSB purchases volume with a low-tier drink decreased by 85.8% per household per week and the amount of sugar consumed from the product decreased by 11.5 g. Products that are not levied experienced a significant change in purchases, were 685.5 ml or the equivalent of 40.2% per household per week in March 2019.

Overall, the purchasing pattern of SSB has increased to 188.8 ml per household per week, while the average consumption of sugar from SSB has reportedly decreased by 2.7% per household per week or the equivalent of 8.0 g after 5 years of tax implementation. Although the number of purchases of products that are not levied has increased, this has not reduced the amount of sugar consumed from SSB products with low and high-tier drinks. This is thought to be caused by consumer preference factors that are difficult to control. Rogers et al. (55) also highlighted that the average sugar content in no-levy drinks (<5 g/100 ml) paradoxically increased. On the other hand, the SSB industry has also changed the sugar concentration in products with a sugar content of >8 g/100 ml to below the sugar concentration threshold, so that these products will not be taxed.

The SSB tax does not significantly reduce Non-communicable Diseases (NCDs) in America due to a few reasons. Firstly, the applied tax rate was very low, as noted by Kim and Kawachi (51) and Powell et al. (52). A low tax rate does not impact the high-income population significantly, as highlighted by studies from Basu et al. (30), Sharma et al. (57), and Kao et al. (43). Secondly, the increase in calorie consumption from substitute products, like milk, has led to obesity and overweight issues persisting, as seen in studies by Escobar et al. (21), Fletcher et al. (48), and Manyema et al. (41). Lastly, changes in SSB consumption in different age groups were influenced by individual behaviors. Adults can choose differently with price increases, while children and teenagers base their choices on parental preferences, especially if the family has a lower income, as shown by Sowa et al. (36). Additionally, not all states in the United States implemented taxes between 1989 and 2006, preventing a significant nationwide decline in obesity and overweight rates.

### 3.3 Is the SSB tax effective to reduce the prevalence of NCDs?

In its implementation process, the tax could not immediately limit only the consumption of SSB in the form of soft drinks. The calorie contribution from SSB needed to be considered so that the implementation of the tax was targeted, effective, and efficient in reducing the prevalence of NCDs. Several countries that had implemented taxes provided insights that SSB taxes were less effective when implemented because there had been a significant reduction in sugar consumption even long before the tax was applied. For example, the consumption pattern of added sugar in countries that conducted modeling simulations such as South Africa, America, Germany, Brazil, Australia, the Philippines, Ireland, Mexico, Thailand, and the UK exceeded the recommended consumption limit set by WHO, which was 50 g *per capita* per day.

This consumption amount contributed at least more than 10% of daily calorie intake. This was in line with the statement of Keller and Bucher Della Torre (6), which stated that SSB contributed 10–15% of daily calories for children and adolescents. This statement became the main assumption in this study to assess the effectiveness of SSB tax implementation. If the calorie consumption from SSB was less than 10%, then a review was necessary before implementing the tax, such as considering sources of added sugar from other food categories that could contribute to calories. The consumption pattern of added sugar, whether sourced from SSB or other beverages, was able to provide results consistent with the modeling simulation that SSB taxes could affect the prevalence of NCDs in those countries.

**Figure 2:**
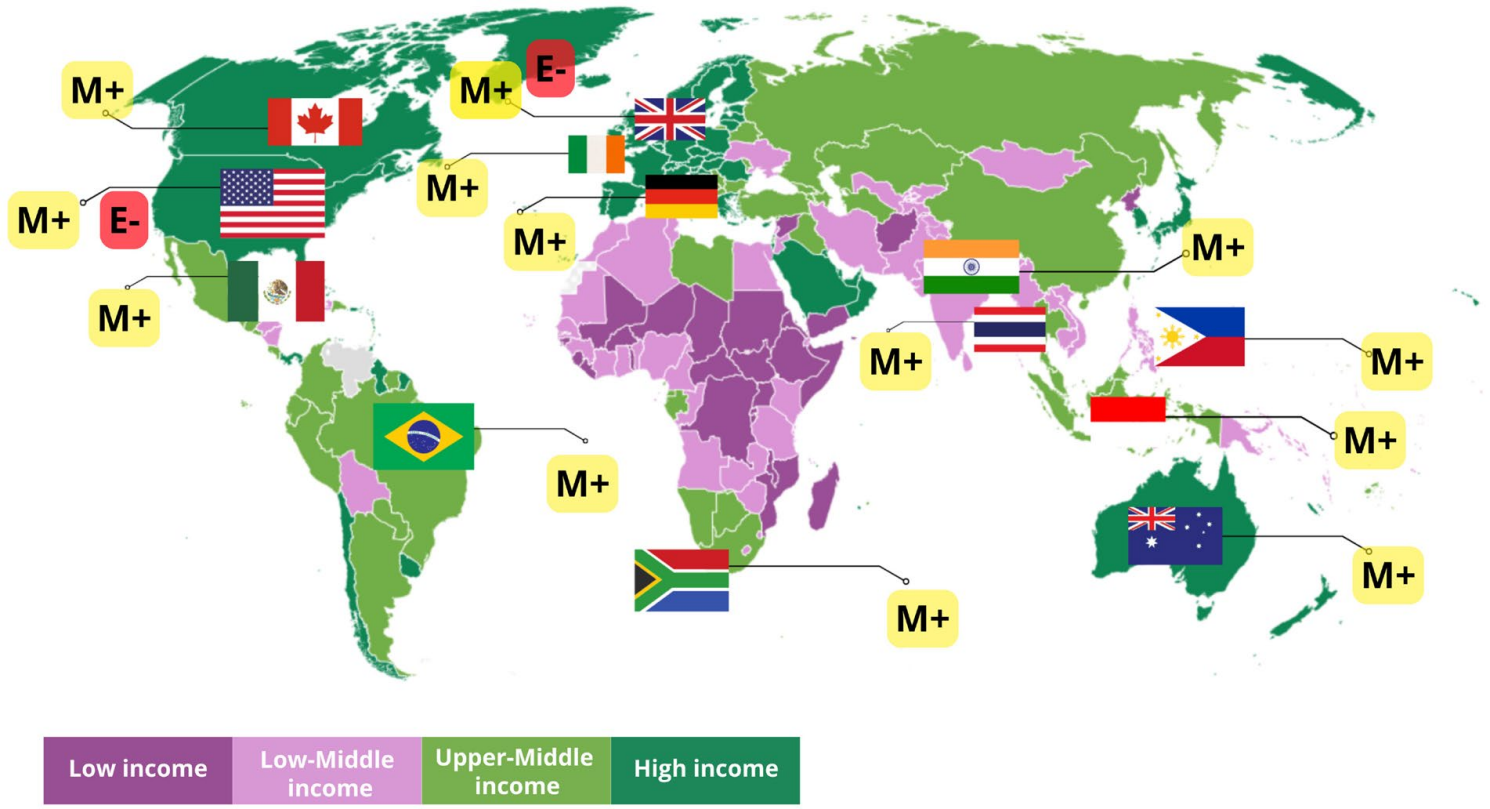
World Bank Group country classification by income that has implemented a tax on sweetened beverages based on modeling and empirical evidence. [M+] shows that the SSB tax in the country has positive implications for the prevalence of NCD based on modeling. [E−] indicates that implementing the SSB tax in the country does not affect the prevalence of NCD based on empirical evidence. Source: World Bank Income Classification FY24 (Worldbank.Org).

In Figure 2, South Africa is an Upper-Middle Income Country (UMIC) with an SSB consumption amount of 518.99 ml/capita/day, SSB contributed 8.76–11% of daily calories for the age group 18–39 years, equivalent to 43.8 g/capita/day (58, 59). Meanwhile, the consumption of added sugar in South Africa *per capita* per day reached 51.32 g, which could contribute 12.93% of daily calories (60). If the own-price elasticity value in South Africa was −1.2, meaning that every 10% price increase due to the tax resulted in a 12% decrease in purchases, then the estimated effectiveness of SSB tax implementation conducted by Manyema et al. (41), and Saxena et al. (34) showed that SSB tax implementation was very effective and efficient for South Africa.

According to USDA (61), the sugar-sweetened beverage industry in South Africa responded to price increases due to the tax by reformulating 30% from 3.1 g/100 ml to 2.4 g/100 ml, but in 2020, when the sugar content reformulation started to stabilize, sugar consumption increased again to 150 thousand tons (still lower compared to before the tax). In another study, it was stated that the decrease in sugar consumption of taxed beverages occurred from 28.8 g/capita/day to 19.8 g/capita/day. However, the consumption of sugar from untaxed substitute products increased by 5.3 g/capita/day (58). Along with the sugar content reformulation, consumers were reported to have decreased interest in reformulated SSB. It could be said that with the increase in sugar consumption again, the SSB tax affected the reduction of SSB purchases but increased the consumption of substitute beverages that still contained high sugar. This caused no effect of the SSB tax on the prevalence of obesity and diabetes in South Africa. Additionally, sugar consumption in South Africa was estimated to have decreased from 1990, which was 40 kg/capita/year to ±33 g/capita/year in 2018 (62).

The simulation in Australia used the assumption of a 6.3% decrease in purchases for every 10% price increase due to the tax (31). This assumption was too low if the added sugar consumption from SSB ranged from 5 to 28 g per day, contributing at least 10–34% of daily calorie intake (63). The estimation results showed that the effectiveness of SSB tax contributed to reducing the prevalence of NCDs. However, according to data, obesity rates in Australia continued to increase (64). At least 33% of the male population was obese in Australia in 2022. This figure was still unaffected by the SSB tax because Australia had not yet implemented an SSB tax. With increasing obesity rates, the SSB tax had the potential to be implemented in Australia to reduce consumption, purchase volume, and also state revenue. If the condition in Australia was compared to America, both are High-Income Countries (HIC) (see Figure 2), where the consumption of added sugar from SSB was equivalent to that in Australia (11–28 g) and contributed 20–37% of daily calorie intake (65) with own-price elasticity values ranging from −0.8 to −1.47 (66, 67), it resulted in a very ineffective estimate due to the low tax rate implemented (38). The tax rate in America needed to be reviewed again by considering the rate recommended by WHO, which was 20% or changed to a volumetric rate.

For example, in San Francisco and Seattle, which implemented SSB taxes in 2018, the tax was ineffective when looking at the obesity rates in 2020, which reached 41.8% for adult women and 42.2% for adult men (64). In these two states, the type of tax implemented was a specific tax, meaning it targeted all types of beverages containing >25 calories per 12 ounces of sugar. It could be said that American consumer preferences did not change, and there was no reduction in the purchase volume of sugar-sweetened beverages, so a review of factors related to sugar and sugary beverage consumption patterns was necessary. According to USDA data (2003–2006), when the SSB tax was implemented, there was an overlooked substitute product variable, which was milk. Milk contributed at least 13.45% of daily calories in America and increased its contribution by 0.6% in adults and 1.7% in children when the SSB tax was implemented, while the calorie contribution from SSB only decreased by 48.8 calories or equivalent to −2.4% in children and 36.8 calories or −1.84% (68).

Brazil was one of the countries that had long implemented an SSB tax, since 1965. The added sugar consumption sourced from SSB in Brazil contributed about 10.7% of calories, where soft drinks only contributed 2.8% of calories (69, 70). However, coffee consumption in Brazil could contribute at least 6.8% of daily calorie intake. Based on simulations, the implementation of the SSB tax showed effectiveness in reducing the prevalence of NCDs with the assumption of own-price elasticity values of soft drinks ranging from −1.19 to −1.36 (39, 71). With the assumption of high own-price elasticity values, the purchase volume of SSB was estimated to decrease from 90.9 L/capita/year to 78.54 L/capita/year (72). Unfortunately, the obesity prevalence in Brazil continued to increase over time (21.8% in 2019) (64). This was suspected to be due to the reduction of the SSB tax rate in 2017 to 4%, which provided evidence that the SSB tax was ineffective in reducing obesity prevalence in Brazil.

In Mexico, the daily volume of SSB purchases was 28.5 g lower compared to Brazil. In line with the conditions in Brazil as a UMIC, soft drinks consumed more than 7 times per week (335 ml per consumption) in Mexico contributed 10.7% of calories (73). In 2014, since the implementation of the SSB tax, there was a 6% decrease in purchase volume. According to the research by Barrientos-Gutierrez et al. (32), the demand for SSB in Mexico after the price increase due to the tax could be seen across economic strata. The low-income group experienced a 17.4% decrease in SSB demand for every 10% price increase, while the middle- and high-income groups experienced decreases of 13.1 and 6.8%, respectively. This was suspected to be due to the ease of accessing SSB. The lower the economic strata, the higher the decrease in the volume of SSB purchases after being taxed.

Considering the contribution of calorie intake and purchase volume, the SSB tax was very appropriate to be implemented in Mexico to encourage a reduction in added sugar consumption associated with NCDs. The effectiveness of the SSB tax has been proven to reduce the prevalence of NCDs in Mexico. According to simulations conducted by Barrientos-Gutierrez et al. (32), the prevalence of obesity decreased by 2.54% and there were 267 fewer new cases of T2DM if prices increased by 20%. However, this was contrary to the data from the Mexican government, where obesity rates among adults and children continued to increase (64). Therefore, consumer soda consumption preferences were not influenced by the SSB tax, making the tax ineffective in reducing obesity prevalence, but effective in reducing purchase volume and state revenue. Although there was a decrease in sugar and HFCS consumption in 2022, sugar and HFCS consumption in Mexico had actually been decreasing since 2006, exactly 8 years before the implementation of the SSB tax (74).

Several countries in Southeast Asia (see Figure 2) that are included in Lower-Middle-Income Countries (LMIC) such as the Philippines and Upper-Middle-Income-Countries (UMIC) such as Indonesia and Thailand show different patterns of added sugar consumption from SSB. A total of 4,846,000 L of SSB are consumed annually by the Filipino people, which contributes to a daily calorie intake of 5.70–16.60% (75). This intake is estimated to decrease by 16.6% for every 10% increase in SSB prices (34). The price elasticity value is considered very appropriate and can represent a real decline in the trend in the Philippines. Based on the assumption of daily calorie intake from SSB exceeding the 15% value, modeling conducted by Saxena et al. (34) showed a significant decrease in diabetes-related deaths (5,913 cases), ischemic heart disease (10,339 cases), and 7,950 deaths from stroke over 20 years. This estimate is quite high considering the prevalence of obesity in adult women in the Philippines is only 8.7% and in adult men it is only around 5% (64).

In 2014, the SSB purchasing pattern in Indonesia reached 3,984 billion liters (33). Based on BPS data, Indonesian people tend to consume home-cooked food rather than processed food and beverages (76). When viewed from the increasing need for sugar use, food sources containing added sugar in Indonesia need to be considered. Andarwulan et al. (77) stated that consumption of added sugar can come from home-cooked food, processed food, and ready-to-eat food. This statement is supported by the conditions in Thailand, where sugar consumption that needs to be considered does not only come from SSB, but also from ready-to-eat foods, and home-processed foods (46). The average consumption of added sugar in Indonesia is 34.9–45.8 g/capita/day, with the highest daily consumption occurring in the male group, both school-age children and adults. Consumption, which is predicted to continue to increase, is contributed by the processed food group (±60%) and ready-to-eat foods (±30%). In this case, consumption of processed food and ready-to-eat foods is contributed from packaged SSB and ready-to-drink drinks from contemporary beverage shops and restaurants as much as 14.5–24.5 g/capita/day. This consumption pattern contributes at least 2.87% of calorie intake in children, 3.50% in adolescents, and 4.56% in adults. These results are in line with research by Atmarita et al. (78), that added sugar only contributes around 3.5–5.25% of daily calorie intake. Although classified as an Upper-Middle-Income Country, the amount of added sugar consumption is lower when compared to the Philippines and Thailand, as well as countries in the Americas and Europe. Therefore, there is an assumption based on the assumption that the daily calorie intake from SSB of 10–15% as a reference for the implementation of SSB tax is not well targeted for implementation in Indonesia.

This is thought to be due to the high consumption of added sugar from other food category sources, such as snacks which contribute 22.17% of the total added sugar intake in adults (77). However, the modeling results show the effectiveness of the implementation of the SSB tax. The database used for modeling is assumed to be the same as the conditions in Mexico, Ecuador, and New Zealand due to data limitations, so if examined further there is an overestimation of the modeling that has been carried out by Bourke and Veerman (33) due to only focusing on soft drink consumption with data that does not represent consumption patterns in Indonesia, because the calorie intake from SSB in Indonesia is no more than 5%. In addition, it does not consider substitute products as stated by Andarwulan et al. (77) such as dairy products, snacks, and bread and the estimated price elasticity value itself is too high, namely −1.33 for low-income groups and −1.20 for high-income groups. This can cause new problems where taxes increase the body’s calorie intake from substitute products which increases BMI (39). Based on this evaluation, the implementation of taxes in countries with ‘low-calorie intake from SSB’, it is necessary to conduct a study considering food products that contribute to added sugar sources so that taxes can be targeted effectively and efficiently.

Our findings show that based on modeling, the implementation of SSB taxes in LMIC and UMIC requires more studies related to the consumption patterns of each country so that the SSB tax parameters used will represent LMIC and UMIC well, particularly in addressing public health challenges associated with non-communicable diseases (NCDs). SSB taxes are linked to reducing the purchased volume of SSB and the consumption of added sugar from SSB. SSB tax also increases the revenue that can be reinvested into the overall health system LMIC. However, based on empirical studies, the implementation of the SSB tax is ineffective in reducing the prevalence of NCDs due to changes in sugar consumption patterns that have decreased long before the tax was implemented, such as in South Africa, Brazil, and Mexico, as well as shifts in SSB purchasing patterns by consumers who are not affected by the tax, as well as SSB consumption patterns.

Policymakers face challenges including the resistance of beverages industries. Industries that are directly affected by the implementation of tax will avoid losses due to shifts in purchasing patterns towards ‘cheaper’ products or products that are not taxed by reformulating, which is good for supporting the healthy lifestyle movement. However, along with the reformulation, the SSB tax may be able to show implications for the consumption of high-calorie substitute products, as happened in South Africa, America, and Indonesia. The increasing consumption of high-calorie substitute products shows that further studies considering the recommendations for the implementation of a ‘single nutrient’ tax by WHO (79) where the single nutrient referred to here is ‘sugar’ in general must be considered. This recommendation is intended to expand the target of products that use sugar such as snacks, drinks, and food served in restaurants, and so on (77), so that there will be an increase in prices for all products containing sugar which will drive a decrease in the volume of purchases and the number of calories consumed from sugary products without exception.

The implementation of SSB tax has a very high urgency related to the formation of community consumption patterns, especially in LMIC and UMIC children and adolescents where SSB consumption preferences are no longer influenced by parents but are also influenced by social media, so a comprehensive policy is needed to accelerate the achievement of a decrease in NCD prevalence. This comprehensive policy needs to be supported through multiple actions consisting of (1) tax implementation, (2) education and promotion of a healthy lifestyle from an early age, so that the consumption of sugary food will not become a habit and also take the regulatory action to reduce marketing of SSB in social media and activities, particularly to children should be considered, (3) Prioritized specific guidelines for healthy beverages consumption in dietary recommendation, and (4) reformulation of sugar concentration in SSB.

## 4 Limitation

Our study had several limitations. The variety of population data and differences in attributes given to each study make the data incapable of meta-analysis. We may also have missed relevant articles in our search. However, we have applied inclusion/exclusion criteria that allow us to include a range of studies, resulting in a comprehensive commentary on the state of science and allowing us to identify important considerations for simulation modeling as well as future implementation of SSB policy.

## 5 Conclusion

The impact of implementing a sugar-sweetened beverage (SSB) tax on the reduction of non-communicable disease (NCD) prevalence has shown positive results in modeling simulations by the fulfillment of the main assumption, namely daily calorie consumption from SSB ranging from 10–15%; and the tax design in the form of tax rates, tax parameters and assumptions given following WHO recommendations; while based on empirical evidence, there is no significant effect of the SSB tax, due to the low rate which is not following WHO recommendations, and the failure to fulfill the assumptions of substitute products, physical activity, and so on, so that it has limited data. Based on the study conducted in this study, the SSB tax is not effective in reducing the prevalence of NCDs due to consumer preferences that have not changed. However, the results of the study also show that the SSB tax plays a role in limiting consumer autonomy to purchase SSB so that the consumption of free sugar and added sugar from SSB will decrease. In addition, SSB can increase state revenue. Multi-actions are needed to support the sustainability of the implementation of the SSB tax, including education and promotion of healthy lifestyles and encouragement to reformulate SSB products by industry. Modeling that provides a wide range of results is the Proportional Multi-State Life Table combined with Cost-Effectiveness Analysis. The estimation results should then be evaluated for implementation according to WHO recommendations in the form of an ad valorem tax (20%), volumetric tax, or specific tax.

## Data Availability

The original contributions presented in the study are included in the article/Supplementary material, further inquiries can be directed to the corresponding author.

## Glossary

BMI: Body mass index
CASP: Critical appraisal skill program
CRAM: Comparative risk assessment analysis
CVD: Cardiovascular disease
DALYs: Disability-adjusted life year
HALYs: Health-adjusted life year
HOMA-IR: Homeostatic model assessment for insulin resistance
IHD: Ischemic heart disease
LMIC: Low-middle-income country
MI: Myocardial infarction
NCDs: Non-communicable diseases
PMSLT: Proportional multi-state life table
Q1: 1^st^ Quintile (Low-income group)
Q5: 5^th^ Quintile (High-income group)
QALYs: Quality-adjusted life year
QoL: Quality of life
SSB: Sugar-sweetened beverages
T2DM: Type-2 diabetes mellitus
UMIC: Upper-middle-income country
