# Supplementary material for: Modeling and empirical evidence of the impact of implementation of sugar sweetened-beverages tax to reduce non-communicable diseases prevalence: a systematic review
Source: Front Nutr. 2024 Oct 11;11:1448300. doi: 10.3389/fnut.2024.1448300 (PMC11502358; doi:10.3389/fnut.2024.1448300)
Supplement: Supplementary file 1 [file Table_1.docx]

**Supplementary Material**

**Supplementary Table 1. Previous Review and Meta-analysis Studies on SSB Tax**

| Author/year/year | Study characteristic | Study aim | Product | Methods used | The main finding |
| --- | --- | --- | --- | --- | --- |
| [1] | Systematic review | To explore SSB taxation influences and impacts systematically. | SSB | - Scoping review approach to identify SSB taxation influences and impacts. - Developed a feedback-oriented conceptual framework for SSB tax evaluation. | - Rare use of systems thinking in SSB taxation evaluations. - Feedback-oriented framework for SSB tax implications. |
| [2] | Systematic review & Meta-analysis | To assess the outcomes of implemented sugar-sweetened beverage (SSB) taxes globally. | SSB | - Assessment of methodological rigor and data limitations for confounders. - Extraction of main effect size per outcome from each article | - Impact of taxes on soda consumption and health outcomes assessed. - Methodological rigor and data limitations evaluated in the research. |
| [3] | Systematic review | Evaluate the health effects of 100% fruit juice consumption in children and adults.  Assess associations with chronic health conditions like cardiometabolic outcomes and caries. | Fruit juice | - Searched PubMed and Cochrane for systematic reviews and meta-analyses. - Conducted meta-analysis and extraction of effec size per outcome from each article | - 100% fruit juice has no adverse health effects except for tooth decay. - Guidelines recommend moderate consumption of 100% fruit juice. |
| [4] | Systematic review | Evaluate the effectiveness of fiscal policies on food and non-alcoholic beverages. | non-alcoholic beverages | Searched by Title and abstract screening | Taxes and subsidies change consumption, but substitution likely occurs. |
| [5] | Systematic review & Meta-analysis | Evaluate the impact of SSB taxes on consumption, obesity, and BMI.  Consider the potential switch to alternative drinks due to tax.  Assess price elasticity and effects on overweight and obesity. | SSB | - Systematic literature review conducted for SSB-related articles from 2000-2013. - Used databases like Pubmed, Google Scholar, and EconLit. - Inclusion criteria: English articles on SSB price impact on consumption. - Excluded articles without standard error or unclear SSB definitions. | - Taxing SSBs may reduce obesity and BMI. - Higher SSB prices lead to lower demand, affecting consumption levels. |
| [6] | Systematic review | Evaluate policy effectiveness in improving healthy food consumption to prevent NCDs. | Sugary drinks | - Screening of 1805 publications for systematic and non-systematic reviews. - Categorization of data using a seven-part framework. - Inclusion of 58 systematic and non-systematic reviews. - Used PICOS approach for study assessment. | - Multi-component interventions and reformulation are effective in promoting healthy diets. - Taxes and subsidies reduce sugary drink consumption and increase fruit/vegetable intake. - Banning junk food ads to children decreases calorie intake effectively. |
| [7] | Systematic review | Evaluate SSB tax impact on consumption, purchase, and obesity prevalence.  Assess differences in effects based on countries' income classification. | SSB | - Systematic review following PRISMA guidelines. - Quality criteria applied for study selection. - Critical appraisal tool used for study quality assessment. | - SSB tax effective in reducing purchase, consumption, and obesity prevalence. - Higher tax rate leads to a greater decrease in overweight/obesity prevalence. |
| [8] | Systematic review | Assess SSB taxation effects on consumption, and obesity prevalence in MICs.  Examine price changes, demand shifts, and substitution effects post-taxation.  Evaluate SSB tax impact on net energy intake and obesity outcomes. | SSB | - Followed PRISMA guidelines for systematic reviews. - Reviewed studies from MICs on SSB taxation effectiveness. - Extracted key data from studies on SSB consumption and obesity. | - Taxing SSBs reduces consumption and obesity prevalence in MICs. - SSB tax increases prices, leading to lower net energy intake. - Milk is a likely substitute for SSBs post-taxation. |
| [9] | Systematic review | Review global SSB consumption and its relation to obesity, and cardiovascular disease.  Assess the effectiveness of soda taxes in reducing SSB consumption. | SSB | - Literature review on SSB consumption and cardiovascular disease risks. - Evaluation of operational results of soda tax policies in countries. | Taxing SSBs effectively reduces consumption, aiding obesity and heart health. |
| [10] | Systematic review | Evaluate the impact of HFSS food taxes on dietary intake and obesity.  Analyze the effects of tax base, rate, and equity on behavior. | High in Fat, Salt, and Sugar | - Linearized almost ideal demand system used for estimation strategy. - Fixed-effects models with three-stage least squares for estimation strategy. | - Experimental HFSS food taxes effectively reduce sales, purchases, and intake. - Studies showed a 4.0% reduction in saturated fat intake. |
| [11] | Systematic review & Meta-analysis | Analyze the impact of local U.S. sugar-sweetened beverage taxes on demand.  Evaluate the price elasticity of demand post-implementation of SSB taxes. | SSB | - Meta-analysis of 26 estimates from 19 studies on SSB taxes. - Used peer-reviewed studies and governmental reports from 2015-2021. - Conducted searches in PubMed, Web of Science, EconLit, and Google Scholar. - Included studies with quantity measures, excluding sales in dollars. | - Local U.S. SSB taxes led to a 20% reduction in demand. - Price elasticity of demand was -1.5 on average. - Cross-border shopping offset a quarter of the demand reduction. |
| [12] | Systematic review | Synthesize evidence on SSB tax impact on consumption, purchase, sales.  Evaluate the impact of taxes on SSBs in real-world scenarios. | SSB | - Followed PRISMA guidelines for systematic reviews. - Included naturalistic and virtual/experimental studies on SSB tax impact. | - Taxes reduce SSB purchases and increase healthy beverage consumption. - SSB taxes lower calorie and sugar intake, impacting diet quality. |
| [13] | Systematic review | Summarize global SSB tax laws.  Assess the impact on obesity, diabetes, and overweight prevalence rates. | SSB | - Systematic scoping review on SSB taxation laws. - Interrupted time series analysis on 17 countries with taxation. - Random-effects meta-regression to assess law impact on health outcomes. | - Taxation on SSBs associated with reduced overweight and obesity rates. - Meta-regression assessed factors affecting the impact of SSB taxation. - Level or slope reduction in overweight and obesity prevalence among youth. |
| [14] | Systematic review | To review the impact of taxation on sugar-sweetened beverage purchases.  To assess the effectiveness of taxes in promoting dietary changes. | SSB | - PRISMA guidelines followed for systematic review methodology. - Five databases were searched systematically from 2000 to May 2017. - Inclusion criteria: studies on SSB price impact using empirical data. - Quality assessment checklist used for all studies included. - Multispectral strategies like taxation of unhealthy foods are considered. | - Taxation reduces the purchase of high-sugar products and calorie intake. - The SSB excise tax in Berkeley led to decreased SSB sales. |
| [15] | Systematic review & Meta-analysis | Evaluate real-world SSB tax impact on purchases and dietary intake.  Examine taxed and untaxed beverage consumption outcomes. | SSB | - Followed PRISMA guidelines for review. - Conducted title screening, data extraction, and bias assessment independently. | - 10% SSB tax led to 10% decline in purchases and intake. - SSB taxes effectively reduced purchases and dietary intake in real-world settings. |
| [16] | Systematic review | Identify methodological challenges in economic evaluations of SSB taxes.  Examine how challenges were addressed in economic evaluations of SSB taxes. | SSB | - Data extraction form developed to collate evaluation information. - Narrative synthesis approach used to analyze data. | - Economic evaluations of SSB taxes face methodological challenges in public health. - Challenges include measuring effects, valuing outcomes, assessing costs, and equity. |
| [17] | Systematic review | Analyze health taxes' impact on consumption, health outcomes, and revenue generation.  Examine the effectiveness of health taxes in reducing unhealthy product consumption. | SSB | - Systematic search for empirical literature on health-damaging product taxes. - Included studies, experiments, public opinion surveys, and qualitative approaches. | - High tax rates on SSBs positively impact health behaviors and outcomes. - Clear prioritization of objectives is crucial when designing taxes. |

HFSS: High in Fat, Salt, and Sugar; MIC: Middle-income country; SSB: Sugar Sweetened-Beverages

**Supplementary Table 2. Definition of fiscal parameters**

| **Term** | **Definition** |
| --- | --- |
| *Own-price elasticities* | A parameter to see consumer responses to changes in SSB prices due to the implementation of the tax. The value is always negative, which indicates that demand for the product will decrease as the price increases. If the value is ≥ 1, then market demand is elastic, but if < 1, market demand is called inelastic.  Example: the value of own-price elasticities is -1.59 and the tax rate is 10%, so for every product that experiences an increase of 10%, SSB purchases will experience a decrease of 15.9%. |
| *Cross-price elasticities* | A parameter to see the response of consumers in purchasing substitute products to changes in SSB prices due to the implementation of the tax.  For example: if a cross-price elasticity value of 0.9 and a tax rate of 10%, then every time the product price increases by 10%, purchases of substitute products will increase by 9%. |
| *Pass-on rate* | The tax margin value will be passed to consumers from the food production chain. Depending on the supplier, manufacturer, retailer, and demand-supply chain, it can be less than, equal to, or greater than 100%. |
| *Ad valorem tax* | The tax uses rates in percentage form.  Example: Australia applies a tax rate for soft drinks of 15%. |
| *Specific tax* | The tax will increase as the sugar content in SSB increases. The determination of specific tax rates depends on consumption patterns in each country.  Example: Thailand applies The SSB tax with sugar content >14 g/100 ml subject to tax at a high rate. |
| *Volumetric tax* | Taxes are applied based on a certain volume of a product.  Example: Mexico sets 1 peso per 1 liter of SSB. |

**Supplementary Table 3. Quality Study using CASP 2018**

| **Reference** | **CASP for Economic Evaluation** | | | | | | | | | | | | |
| --- | --- | --- | --- | --- | --- | --- | --- | --- | --- | --- | --- | --- | --- |
|  | **Q1** | **Q2** | **Q3** | **Q4** | **Q5** | **Q6** | **Q7** | **Q8** | **Q9** | **Q10** | **Q11** | **Q12** | **Total** |
| [18] | **✓** | **✓** | **✓** | **✓** |  |  | **✓** |  |  | **✓** |  | **✓** | **7** |
| [19] | **✓** | **✓** |  | **✓** |  |  | **✓** |  | **✓** | **✓** |  | **✓** | **7** |
| [20] | **✓** | **✓** | **✓** | **✓** |  |  | **✓** |  |  | **✓** |  | **✓** | **7** |
| [21] | **✓** | **✓** |  | **✓** |  |  |  | **✓** | **✓** | **✓** |  | **✓** | **7** |
| [22] | **✓** | **✓** |  | **✓** |  |  |  | **✓** | **✓** | **✓** |  | **✓** | **7** |
| [23] | **✓** | **✓** |  | **✓** |  |  |  | **✓** | **✓** | **✓** |  | **✓** | **7** |
| [24] | **✓** | **✓** | **✓** | **✓** |  |  | **✓** |  | **✓** | **✓** | **✓** | **✓** | 9 |
| [25] |  | **✓** | **✓** | **✓** |  |  | **✓** |  | **✓** | **✓** | **✓** | **✓** | 8 |
| [26] |  | **✓** | **✓** | **✓** |  |  | **✓** | **✓** | **✓** | **✓** | **✓** | **✓** | 9 |
| [27] | **✓** | **✓** | **✓** | **✓** |  |  | **✓** |  |  | **✓** |  | **✓** | 7 |
| [28] |  | **✓** | **✓** | **✓** |  |  | **✓** |  | **✓** | **✓** | **✓** | **✓** | 8 |
| [29] | **✓** | **✓** | **✓** | **✓** |  |  | **✓** |  | **✓** | **✓** | **✓** | **✓** | 9 |
| [30] | **✓** | **✓** | **✓** | **✓** |  |  | **✓** |  | **✓** | **✓** | **✓** | **✓** | 9 |
| [31] | **✓** | **✓** | **✓** | **✓** | **✓** | **✓** | **✓** | **✓** | **✓** | **✓** | **✓** | **✓** | 12 |
| [32] | **✓** | **✓** | **✓** | **✓** | **✓** | **✓** | **✓** | **✓** | **✓** | **✓** | **✓** | **✓** | 12 |
| [33] |  | **✓** | **✓** | **✓** |  |  | **✓** | **✓** | **✓** | **✓** | **✓** | **✓** | 9 |
| [34] | **✓** | **✓** | **✓** | **✓** |  |  |  | **✓** | **✓** | **✓** | **✓** | **✓** | 9 |
| [35] | **✓** | **✓** | **✓** | **✓** |  |  | **✓** |  | **✓** | **✓** | **✓** | **✓** | 9 |
| [36] | **✓** | **✓** | **✓** | **✓** | **✓** | **✓** | **✓** | **✓** | **✓** | **✓** | **✓** | **✓** | 12 |
| [37] | **✓** | **✓** | **✓** | **✓** |  |  | **✓** | **✓** | **✓** | **✓** | **✓** | **✓** | 10 |
| [38] | **✓** | **✓** | **✓** | **✓** |  |  | **✓** | **✓** | **✓** | **✓** | **✓** | **✓** | 10 |
| [39] | **✓** | **✓** | **✓** | **✓** |  | **✓** | **✓** | **✓** | **✓** | **✓** | **✓** | **✓** | 11 |
| [40] | **✓** | **✓** | **✓** | **✓** | **✓** | **✓** | **✓** | **✓** | **✓** | **✓** | **✓** | **✓** | 12 |
| [41] | **✓** | **✓** | **✓** | **✓** | **✓** | **✓** | **✓** | **✓** | **✓** | **✓** | **✓** | **✓** | 12 |
| [42] |  | **✓** | **✓** | **✓** |  |  | **✓** |  | **✓** | **✓** | **✓** | **✓** | 8 |
| [43] | **✓** | **✓** | **✓** | **✓** |  |  | **✓** | **✓** |  | **✓** | **✓** | **✓** | 9 |
| [44] |  | **✓** | **✓** | **✓** |  |  | **✓** |  | **✓** | **✓** | **✓** | **✓** | 8 |
| [45] | **✓** | **✓** | **✓** | **✓** |  |  | **✓** |  | **✓** | **✓** | **✓** | **✓** | 9 |
| [46] | **✓** | **✓** | **✓** | **✓** | **✓** | **✓** | **✓** | **✓** | **✓** | **✓** | **✓** | **✓** | 12 |

Q1. Does the study include all parameters such as time horizon, intervention, objectives, costs, and their consequences?

Q2. Is there a comprehensive definition related to the provided alternatives?

Q3. Does the study show evidence that the given alternatives will be effective? (For example, does the program provide positive impacts compared to negative ones?)

Q4. Are the impacts of the intervention identified, measured, and adequately assessed? (showing the unit value of the existing outcomes)

Q5. Are all essential resources, as well as health costs for each intervention, identified, measured, and credibly assessed? (e.g., values of HALYs, QALYs, DALYs; healthcare costs)

Q6. Can costs and consequences be adjusted for different timeframes (discounted)?

Q7. What are the evaluation results?

Q8. Have additional analyses of alternative consequences and costs been conducted?

Q9. Was sensitivity/uncertainty/validity analysis performed?

Q10. Can the program be implemented in your region?

Q11. Can health costs and intervention costs be adapted in your region?

Q12. Is the program feasible to be carried out in your region?

**Supplementary Table 4. Definition from the model design**

| **Model** | **Definition** | **Strength** | **Limitation** | **Model Assessment** |
| --- | --- | --- | --- | --- |
| Math Simulation | Simulation based on theoretical and mathematical calculations using price elasticity values | - Provides the dynamic interaction of factors and simulates long-term impacts that cannot be assessed through empirical studies - Helps estimate the potential impact of interventions and anticipate the consequences of existing intervention strategies | - Accuracy depends on the validity of the assumptions and the quality of the data used - Requires quite a long time - Difficult to interpret | *Sensitivity analysis* |
| Microsimulation | Computer-based simulation to simulate a set of data in accordance with predetermined probability rules | - Can capture the impact of an intervention on an individual's risk factor proTable, allowing complex relationships between multiple comorbid risk factors to be incorporated into trials. - Able to process large amounts of data | - The level of detail of the model does not align with all predictions because it is random - Bias may occur - costs a lot of money and times | *Validation analysis* |
| Cohort | Models that follow a specific group of individuals have specific experiences or characteristics such as disease, disease severity, and risk exposure over a certain period of time | - Produces very homogeneous data - As a more impactive consideration in decision-making when combined with cost-effectiveness analysis - Capable of capturing the impact of interventions in diverse populations | - Requires collection of data over time - Research costs are expensive - Potential bias impacting the validity of the findings | *Sensitivity Analysis* |
| Proportional Multi-State Life Table | Models used to estimate the impact of preventive interventions on life-years | - Capture the complex health transitions of multiple health conditions - The model considers the presence of different risk factors for causes of death and their transitions - Estimate the impacts of interventions thereby providing information about the impact of interventions on different health outcomes | - The cost of analysis and parameters is quite large because it uses a large model structure - Requires comprehensive, high-quality data - The model relies on complex assumptions - Potential for bias is very high - Interpretation of this model is difficult | *Sensitivity analysis* |
| Cost-effectiveness Analysis | Analysis used to assess the efficiency and benefits of health facility technology for producing health outcomes relative to the costs of different health interventions | - Able to provide direct comparisons of various medical interventions based on their economic efficiency - As information that can assist in the uptake, reimbursement, or coverage of health interventions - More transparent so that it can be taken into consideration by stakeholders | - Inability to compare the same interventions, but with different health outcomes - Depends on the availability, accuracy and quality of data - Does not include equality or socio-economic values - Assumptions must be simplified to estimate costs because they are unable to capture complex data | *Sensitivity analysis* |
| Comparative Risk Assessment | Systematic evaluation of changes in health in a population resulting from exposure to a risk factor or group of risk factors | - Can be applied in various fields and provides relatively accurate results. - Use a systematic approach, so that the burden of disease and potential interventions can be comprehensively evaluated - Provide information about potential preventable health outcomes from existing interventions - Help stakeholders prioritize interventions and resource allocation based on existing health potential - As evidence-based information that can be used for adoption and implementation of interventions | - Accuracy depends on the validity of the assumptions and the quality of the data used | *Validation analysis*  *Sensitivity analysis*  *Uncertainty analysis* |
| Econometric-Epidemiologic | Analysis of the relationship between preventive behavior and disease prevalence that focuses on economic causes and epidemiological consequences arising from the spread of disease that affect public health | - Provide integrated data on expenditure to prevent disease (economic sector) and disease conditions that arise - The data produced is relatively accurate - Allows researchers to explore cause-and-impact relationships between interventions and health outcomes - Able to calculate costs, so that potential maintenance savings can be identified - As information on resource allocation, priority setting, and interventions to maximize population health based on economic efficiency | - Requires quite a long time - Accuracy depends on data quality | *Sensitivity Analysis* |
| System Dynamics Model | the process of identifying a problem by preparing a qualitative hypothesis (qualitative system dynamics) which is supported by a description of a relationship with a flow diagram which is then translated into a quantitative simulation (quantitative system dynamics) in order to obtain reliability values and policy analysis | - Able to describe complex situations and anticipate small changes over time - The information base used is very broad - Able to describe complex conditions with poor data sets | - Cannot run more than one version of a situation in a single analysis even if it captures many changes - Biases can arise due to differences in opinions of various stakeholders in viewing complex situations | *Sensitivity Analysis* |

**Supplementary Table 5. The SSB tax impact estimation on NCDs**

| **Model** | **Reference** | **Time Horizon** | **Population** | **Tax rate** | **Result** | | | | | | | |
| --- | --- | --- | --- | --- | --- | --- | --- | --- | --- | --- | --- | --- |
|  |  |  |  |  | **BMI** | **Overweight** | **Obesity** | **T2DM** | **CVD** | **DMFT** | **CA** | **Stroke** |
| Math simulation | [28] | - | 28,255 individuals | 20% |  |  | -3.8% (95% CI: 0.6%–7.1%) (M)  -2.4% (95% CI: 0.4%–4.4%) (F) |  |  |  |  |  |
| Microsimulation | [29] | 5 years | 7,049 individuals | 20% |  |  | -3.0% (95% CI: 1.6%–5.9%) | -1.6% (95% CI: 1.2%–1.9%) |  |  |  |  |
|  | [30] | 20 years | School-age (6-12 years)  Adolescent (13-18 years) | $\$0.01/ounce$ |  | 0.3 % (6–12 years)    0.1% (13–18 years) | -1.6 % (6–12 years)    -2.4% (13–18 years) |  |  |  |  |  |
|  | [34] | 35 years | 96,031 adults individuals | 10% and 20% | Tax rate 10%: -0.15$\pm$0.55 Kg/m^2^/month  Tax rate 20%: -0.31$\pm$0.01 Kg/m^2^/month |  | -2.54% (overall)  Tax rate 10%: 134 cases  Tax rate 20%: 267 cases |  |  |  |  |  |
|  | [40] | lifetime | 1,000,000 adults | $ 0.01/ounce |  |  |  |  | MI: 4,494 (95% CI: 2,640 to 6,599)cases/ 1 million adult people |  |  | -60 (95% CI:–81 to 181) cases/ 1 million adult people |
| Proportional Multi-state Life Table | [32] | 20 years | Ages $\geq$ 20 years Australian population 2010 | 20% |  |  | -2.7% (M)  -1.2% (F) | -800 cases/ years  After 25 years 15,875 (95% CI: 7,480–25,050) cases | IHD: -4,426 (95% CI: 3,047 –5,939) cases |  |  | *-*1.118 (95% CI: 521–1,809) cases |
|  | [36] | 25 years | Household  SUSENAS | 20% |  | -2.9% (M)  -1.4% (F) | -7.3% (M)  -3.9% (F) | -63,000 cases (95% CI: -245,067 to -8410) (Q1)  -1,487,000 cases (95% CI: -3,068,603 to -545,913) (Q5) | IHD: -0.07% (95% CI: -1,965 to -1,029) (Q1)  -2.1% (95% CI: -58,765 to -38,724) (Q5) |  |  | -0.07% (-1,384 cases; 95% CI: -1,883 to -943) (Q1)  -2.1% (-44,746 cases; 95% CI: -56,744 to -34,291) (Q5) |
|  | [41] | lifetime | 119,099 adults individuals | 20% |  | -36,000 cases | -400,000 cases | -144,901 cases  -32,600 (Q1)  -24,660 (Q5) | -44,118 cases  -9,632 (Q1)  -8,539 (Q5) |  | -17,740 cases | -5,816 cases |
|  | [46] | Lifetime | Adults Canadian 2015 population | CAD$0.015/oz | -0.18 Kg/m^2^ (M)  -0.14 Kg/m^2^ (F) | -0.91% (M)  -0.68% (F) |  | -72,673 cases | -27,972 cases |  | BC: -1,451(95% CI: 1,186–1,708) cases  TC: -522 (95% CI:477–567) cases  CRC: -233 (95% CI: 223–243) cases |  |
| Cohort | [39] | 10 years | 18,700,000 individuals | 20% |  |  |  |  |  | -3.9 million unit/ 10 years |  |  |
| Comparative risk assessment | [22] | - | Ages $\geq$ 18 years | 1% | -0.003%  (0.0004) | -0.0002% (0.0001) | -0.0001% (0.0000) |  |  |  |  |  |
|  | [24] | 10 years | 22,750 Household | 20% | -1.6 Kg/year  -16 Kg/ 10 years | -45.2% / 10 years | -21.8% /10 years |  |  |  |  |  |
|  | [26] | - | 5,263 Household | 20% |  | 0.9% (95% CI: 0.6% to 1.1% | -1.3% (95% CI: 0.8% to 1.7%) |  |  |  |  |  |
|  | [25] | - | 10,000 Adults | 10% |  | -0.7% (14,380 adults; 95% CI: 9,790 to 12,940) | -1.3% (9,900 adults; 95% CI: 7,750 to 12,940) |  |  |  |  |  |
|  | [33] | *-* | 5,236 Household | *Specific tax* |  |  | 81,594 (3588–182,669; 0.5%). cases/years | 10,861 (3899–18,964; 17,7) cases /years |  | -269,375 (82,211–470,928; 4.4% per 1000 people per year) cases |  |  |
|  | [35] | 10 years | Ages 15-79 | 20% |  | -3%  (1,028,000 person) | -4% (479,000 person) |  |  |  |  |  |
|  | [45] | 20 years | 57,290 Household | 20% |  |  |  | After 10 years:  Male: -37,303 (95% CI: 33,736–41,08)  Female:  -56,757 (95% CI: 52,974– 61,001)  After 20 years:  -8.6% (M)  -12.4% (F) |  |  |  |  |
| Cost effectiveness analysis | [31] | 10 years | Ages $\geq$2 years | $\$0.01/ounce$ | -0.08 (95% CI: 0.03 to 0.20) Kg/m^2^ (adult)  -0.16 (95% CI: 0.06 to 0.37) Kg/m^2^ (adolescent) |  | -0.99% (adult)  -1.38% (adolescent) |  |  |  |  |  |
|  | [37] | 20 years | 98.2 billion | 13% |  |  |  | -299,540 cases | IHD: -40,882 cases |  |  | -19,858 cases |
| Econometric-Epidemiologic | [44] | - | Ages $\geq$3 years | 11%; 20%; 25% | Tax rate 11%: -0.21 (95% CI: -0,22 to -0,21)  Tax rate 20%: -0.40 (95% CI: -0.40 to -0.38)  Tax rate 25%: -0.49 (95% CI: -0.50 to -0.47) |  | Tax rate 11%: -1.73% (95% CI: 1.11% to 2.575)  Tax rate 20%: - 3.83% (95% CI: 2.88% to 4.98%)  Tax rate 25%:-4.91% (95% CI: 3.83% to 6.18%( |  |  |  |  |  |
| System Dynamics Model | [42] | 20 years | Ages $\geq$ 15 years | Specific tax |  |  |  |  |  | 1% |  |  |

F: Female; M: Male

BC: Breast Cancer; BMI: Body Mass Index; CA: Cancer; CRC: Colorectal Cancer; CVD: Cardiovasculardisease; DMFT: Decayed Missing Filled-Teeth; IHD: Ischemic Heart Diseases; MI: Myoicardial Infarction; TC: Tyroid Cancer; T2DM: Type-2 Diabetes Mellitus; IHD: Ischemic Heart Disease; MI: Myocardial Infarction; Q1: Quintile 1; Q5: Quintile 5

**Supplementary Table 6. Simulation modeling design methods (N=22)**

| Variable | | n | % |
| --- | --- | --- | --- |
| **Type of simulation model** | | | |
|  | Math simulation | 1 | 5% |
|  | Microsimulation | 4 | 18% |
|  | Cohort modeling | 1 | 5% |
|  | Proportional Multistate life table | 4 | 18% |
|  | Cost-effectiveness | 3 | 14% |
|  | Comparative Risk Assessment | 7 | 32% |
|  | Econometric epidemiologic | 1 | 5% |
|  | System dynamics models | 1 | 5% |
| **Time horizon** | | | |
|  | 10 years | 4 | 18% |
|  | 20 years | 5 | 23% |
|  | lifetime | 3 | 14% |
|  | Not mentioned | 6 | 27% |
|  | Other (5 years, 25 years, 35 years) | 4 | 18% |
| **Simulation modeling methods details^** | | | |
|  | Modeling logical pathway | 14 | 64% |
|  | Table of parameters used | 12 | 55% |
|  | Explanation of assumption | 22 | 100% |
|  | Model assessment (sensitivity, uncertainty, validity) | 22 | 100% |
|  | Stakeholder role | 5 | 23% |
| **Tax Parameter^** | |  |  |
|  | Own-price elasticities | 20 | 91% |
|  | Cross-price elasticities | 9 | 41% |
|  | Pass-on rate | 20 | 91% |
|  | Tax rate (ad valorem, volumetric, specific tax) | 22 | 100% |
|  | Discount | 6 | 27% |
| **Health Measurement Parameter^** | | | |
|  | BMI | 18 | 82% |
|  | Obesity status | 7 | 32% |
|  | T2DM status | 8 | 36% |
|  | Dental caries status | 3 | 14% |
|  | CVD status | 1 | 5% |
| **Simulated Health Parameter^** | | | |
|  | Daily calories intake | 6 | 27% |
|  | Physical activity | 1 | 5% |
|  | Mortality | 3 | 14% |
|  | Prevalence/incidence/mortality due to cancer | 2 | 9% |
|  | Prevalence/incidence/mortality due to cancer | 1 | 5% |
|  | Reducing sugar consumption | 1 | 5% |
|  | Quality of Life (HALYs, DALYs, QALYs) | 9 | 41% |
|  | Economic benefit (Tax revenue, health cost) | 11 | 50% |

^Each article can have various simulation methods and parameters, so that when the percentages add up they exceed 100%

**Supplementary Table 7. Population and Result from Simulation Models (N=22)**

| **Variable** | | **n** | **%** |
| --- | --- | --- | --- |
| **Modeling Country** | | | |
|  | South Africa | 2 | 9% |
|  | Australia | 2 | 9% |
|  | USA | 5 | 23% |
|  | Canada | 2 | 9% |
|  | UK | 3 | 14% |
|  | Thailand | 2 | 9% |
|  | Brazil | 1 | 4.5% |
|  | Germany | 1 | 4.5% |
|  | Philippines | 1 | 4.5% |
|  | India | 1 | 4.5% |
|  | Indonesia | 1 | 4.5% |
|  | Mexico | 1 | 4.5% |
| **Attributes assigned to the population simulation^** | | | |
|  | Age | 21 | 95% |
|  | Gender | 18 | 82% |
|  | Income | 13 | 59% |
|  | Race and ethnicity | 2 | 9% |
|  | Education | 2 | 9% |
|  | Socioeconomic status | 9 | 41% |
|  | SSB consumption | 4 | 18% |
|  | Physical activity | 1 | 5% |
| **Attributes for result stratification^** | | |  |
|  | Age | 15 | 68% |
|  | Gender | 16 | 73% |
|  | Income | 12 | 55% |
|  | Race and ethnicity | 2 | 9% |
|  | Socioeconomic status | 8 | 36% |
|  | BMI clasification | 2 | 9% |
|  | Health cost | 1 | 5% |
| **Simulation result based on health measured parameters^** | | | |
|  | BMI | 5 | 23% |
|  | Overweight | 8 | 36% |
|  | Obesity | 15 | 68% |
|  | T2DM | 8 | 36% |
|  | Cardiovascular | 6 | 27% |
|  | Dental caries | 3 | 14% |
| **Simulation result based on health simulated parameters^** | | | |
|  | Cancer | 2 | 9% |
|  | Stroke | 5 | 23% |
|  | Mortality | 6 | 27% |
|  | Daily calorie intake | 7 | 32% |
|  | *Quality of life* (DALYs, QALYs, HALYs) | 6 | 27% |
|  | Economic benefit | 11 | 50% |

^ Each article can have various attributes and results so that when the percentages are added up, they exceed 100%

**Supplementary Table 8. The SSB tax impact estimation on Mortality, Daily Calories Intake (DCI), Quality of Life (QoL), and Economic Benefit**

| **Model** | **Reference** | **Time Horizon** | **Tax rate** | **Result** | | | |
| --- | --- | --- | --- | --- | --- | --- | --- |
|  |  |  |  | **Mortality** | **DCI** | **QoL** | **Economic Benefit** |
| Math Simulation | [28] | - | 20% |  | 36 kJ/ day |  |  |
| Microsimulation | [40] | lifetime | $ 0.01/ounce | IHD: 1.540 (95% CI: 995 to 2,118) cases/ million adult people |  | QALYs: 3.4 million | Health cost: US$45 (95% CI: 25.43, 65.04) billion |
| Cohort | [39] | 10 years | 20% |  |  |  | Health cost: AS$666$\pm$189 million |
| Proportional Multi-State Life Table | [36] |  | 20% | IHD: 1600 (95% CI:1,252 to 1986) cases |  | HALYs: 112.000 (95% UI: 73,000–155,000) (M)  56.000 (95% UI: 36,000–76,000) (F); | US$400 million/ years |
|  | [36] | 25 years | 20% |  | 17 kJ/people/day | HALYs:  29% (M) 28% (F) | US$15.1 (95% CI: 13,703 to 17,295) billion/year (Q5) and US 536 (95% CI: 488 to 607) million/year (Q1) |
|  | [41] | lifetime | 20% |  |  | DALYs: 760.000 | USD 1.4 billion/ years |
|  | [46] | lifetime | CAD$0.015/oz | 2189 (95% CI: 1,866 to 2,447) cases |  | DALYs: 2.291.373  QALYs: 1.509.349 | Health cost: $37.548 (95% UI: CAD$34,155, 39,784) million  Tax revenue: $44.016 (95% UI: CAD$43,346, 44,620 million) tax revenue over 80 years. |
| Cost-Effectiveness Analysis | [37] | 20 years | 13% | T2DM: 5.913 cases  IHD: 10.339 cases  Stroke: 7.950 cases |  |  | Health cost: 31.6 billion peso  Tax revenue: 41.0 billion peso/years |
|  | [38] | 20 years | 10% | 8000 cases |  |  | Health cost: US$140 million  Tax revenue: US$450 million  Poverty cases averted: 12.179 cases |
|  | [31] | 10 years | $\$0.01/ounce$ |  | 1.56 (95% CI:1.16, 1.97) kcal/ day (2-19 years)  0.90 (95% CI:0.64, 1.16) kcal/ day (> 19 years) | DALYs: 101.000  QALYs: 871.000 | Health cost: $23.6 (95% UI: $9.33, $54.9) billion  Tax revenue: $1823 - $12.6 (95% UI:$ 8.92, $14.1) billion |
| Comparative Risk Assessment | [24] | 10 years | 20% |  |  |  | $5.8 billion/years |
|  | [26] | - | 20% |  | 16.7 (95% CI: 11.3 to 21.7) kJ/people/day |  | £276m (95% CI £272m to £279m) |
|  | [25] | - | 10% |  | 2.1 (95% CI: 1.7 to 2.6) kcal/people/day |  |  |
|  | [35] | 10 years | 20% |  | 376 kJ/ capita (M; *low income*)  128 kJ/ capita (F; *low income*) |  |  |
|  | [45] | 20 years | 20% | -13.7% or 5,386 (95% UI: 5,074–5,727) (M)    -12.7% or 6,075 (95% UI: 5,649–6,531 (F) |  |  |  |
| Econometric-Epidemiologic | [44] | - | 11%; 20%; 25% |  | 11%: 59 kJ/people/day  20%: 109.6 kJ/people/day  25%: 134.9 kJ/people//day |  |  |

F: Female; M: Male

DCI: Daily Calories Intake; T2DM: Type-2 Diabetes Mellitus; IHD: Ischemic Heart Disease; MI: Myocardial Infarction; HALYs: Health-Adjusted Life Years, is a summary measure of population health commonly used in estimating the burden of disease; DALYs: Disability-Adjusted Life Years, is the total length of time a particular disease incapacitates a person during his or her lifetime; QALYs: Quality-Adjusted Life Years: is a measure of the quantity and quality of life lived by a person based on an analysis of the cost-effectiveness of clinical (or public health) interventions and for improving social welfare; QoL: Quality of Life

**Supplementary Table 9. SSB tax effectiveness methods analysis (N=6)**

| Variable | | n | % |
| --- | --- | --- | --- |
| **Design study** | | | |
|  | *Cross-Sectional* | 1 | 17% |
|  | *Repeated Cross-sectional* | 2 | 33% |
|  | *Longitudinal* | 2 | 33% |
|  | *Longitudinal Comparative Case Study* | 1 | 17% |
| ***Time horizon*** | | | |
|  | 1991 and 1998 | 1 | 17% |
|  | 6 years | 1 | 17% |
|  | 10 years | 1 | 17% |
|  | 17 years | 2 | 33% |
|  | 20 years | 1 | 17% |
| **Method analysis details^** | | | |
|  | Display a table of parameters used | 4 | 67% |
|  | Tax impact analysis using OLS regression | 5 | 83% |
|  | *Model assessment (sensitivity, uncertainty, validity)* | 5 | 83% |
| **Tax parameter^** | |  |  |
|  | *Own-price elasticities* | 2 | 33% |
|  | *Cross-price elasticities* | 1 | 17% |
|  | *Pass-on rate* | 1 | 17% |
|  | Tax rate (*ad valorem*) | 6 | 100% |
| **Health Measurement Parameter** | | | |
|  | BMI | 3 | 50% |
|  | Obesity status | 3 | 50% |
|  | HOMA-IR | 1 | 17% |

^Each article can have various methods and parameters, so that when the percentages add up they exceed 100%

BMI: Body Mass Index; HOMA-IR: Homeostatic Model Assessment for Insulin Resistance

**Supplementary Table 10. Population and result from evidence (N=6)**

| **Variable** | | **n** | **%** |
| --- | --- | --- | --- |
| **Country** | | | |
|  | USA | 6 | 100% |
| **Attributes assigned to the population simulation^** | | | |
|  | Age | 6 | 100% |
|  | Gender | 5 | 83% |
|  | Income | 3 | 50% |
|  | Race and ethnicity | 5 | 83% |
|  | Education | 5 | 83% |
|  | Socioeconomic status | 6 | 100% |
| **Attributes for result stratification^** | | |  |
|  | Age | 6 | 100% |
|  | Gender | 5 | 83% |
|  | Income | 2 | 33% |
|  | Race and ethnicity | 1 | 17% |
|  | Socioeconomic status | 2 | 33% |
|  | BMI Classificatication | 2 | 33% |
|  | Education | 3 | 50% |
| **The results of the impactiveness of taxes on NCDs are based on the health parameters measured^** | | | |
|  | BMI | 5 | 83% |
|  | Overweight | 1 | 17% |
|  | Obesity | 2 | 33% |
|  | Daily calories intake | 2 | 33% |
|  | HOMA-IR | 1 | 17% |

^ Each article can have various attributes and results so that when the percentages are added up, they exceed 100%

BMI: Body Mass Index; HOMA-IR: Homeostatic Model Assessment for Insulin Resistance

**Supplementary Table 11. SSB tax impact on NCDs based on evidence**

| **Author** | **Country** | **Population** | **Design Study** | **Product** | **Tax rate** | **Parameter** | **Health Outcome Target** | **Result** |
| --- | --- | --- | --- | --- | --- | --- | --- | --- |
| [18] | USA | All Ages | Cross-sectional (1991 and 1998) | Soft drinks | 5% | BMI $\geq$ 30 Kg/m^2^ obesity  BMI $\geq$ 25 Kg/m^2^ overweight | Obesity prevalence | There is no significant relationship between SSB excise and reducing the prevalence of obesity ($-$1%) |
| [19] | USA | Adolescent (13-19 years, n=153,673) | Repeated cross-sectional (10 years) | Soft drinks | 4.25% | BMI $\geq$ 30 Kg/m^2^ obesity  BMI $\geq$ 25 Kg/m^2^ overweight | BMI (Kg/m^2^) | There is no significant relationship between state-level SSB excise and adolescent BMI (-0.006%; *p value= 0.09*) with the average BMI being 22.13 Kg/m^2^ |
| [20] | USA | Adolescent and adult (18-30 years, 1985-2006) | Longitudinal study (20 years) | soda | USD 1/2 L bottle soda $\approx$ 37% | BMI $\geq$ 30 Kg/m^2^ obesity  BMI $\geq$ 25 Kg/m^2^ overweight  HOMA IR:  $\leq$ 1, sensitive  $\geq$ 1.9, initial resistance  $\geq2.9, significant$ resistance | Daily calorie intake, BMI, and HOMA-IR | Reduce daily calories intake $-$124 kcal (95% CI: $-$17.50, $-5$.50); BMI $-$1.05 Kg/m^2^ (95% CI: $-$1.80, $-$0.31); HOMA-IR 0.42 (95% CI: $-$0.59, $-$0.31) |
| [21] | USA | Children and adolescents (n=34,000) | Repeated cross-sectional (17 years) | *Soft drinks* | Average rate 2.27% | BMI$\geq$ 95th percentile of the age and gender distribution  *Overweight*:  BMI$\geq$percentile of 85 | BMI (Kg/m^2^) and obesity prevalence | There is no significant impact on reducing BMI (-0.015 Kg/m; *Z score=* 0.016), overweight (-0.002%; 0.0011) and obesity (-0.009%; 0.006) in states that implement or do not implement the tax |
| [23] | USA | Children | Longitudinal study (6 years) | Carbonated SSB | 4.2% | BMI $\geq$ 30 Kg/m^2^ obesity  BMI $\geq$ 25 Kg/m^2^ overweight | BMI (Kg/m^2^) | No significant changes to BMI ($-$0.085% Kg/m^2;^ *p value=* 0.05) |
| [27] | USA | Adult (1989-2006) | Longitudinal Comparative case study (17 years) | Soda | 2.59% | BMI $\geq$ 30 Kg/m^2^ obesity  BMI $\geq$ 25 Kg/m^2^ overweight | Daily calorie intake and BMI (Kg/m^2^), | There is no significant relationship between SSB tax and non-linear impacts; Every 1% increase in the tax rate increases the calorie intake of substitute products by 7.5 kcal/day (0.05<0.1); BMI $-$0.007 Kg/m^2^ (0.937> 0.1); and daily calories intake 1.56 kcal/day (0.52 > 0.1) |
| [43] | UK | Adult (n= 22.091, March 2014-March 2019) | Controlled interrupted time series analysis | Soft drinks | *Specific tax*  GBP 0.24/L for SSB with sugar content ≥ 8 g/ 100 ml; GBP 0.18/L for SSB with sugar content ≥5 to <8 g/100 ml; GBP 0 (without tax) for SSB with sugar content $<$5 g/ 100 ml | Average purchase volume per household per week | Volume of soft drink purchases and sugar content of soft drinks | After the tax, the purchasing pattern of high-tier drinks decreased by 37.8% per household per week and sugar consumption from SSB decreased by 16.2 g. SSB purchases volume with a low-tier drink decreased by 85.8% per household per week and the amount of sugar consumed from the product decreased by 11.5 g. Products that are not levied experienced a significant change in purchases, were 685.5 ml or the equivalent of 40.2% per household per week in March 2019 |

BMI: Body Mass Index; GBP: British Pound sterling; HOMA-IR: Homeostatic Model Assessment for Insulin Resistance

**Reference**

[1] M. Alvarado *et al.*, “A systematic scoping review evaluating sugar-sweetened beverage taxation from a systems perspective,” *Nat. Food*, vol. 4, no. 11, pp. 986–995, 2023, doi: 10.1038/s43016-023-00856-0.

[2] T. Andreyeva, K. Marple, S. Marinello, T. E. Moore, and L. M. Powell, “Outcomes Following Taxation of Sugar-Sweetened Beverages: A Systematic Review and Meta-analysis,” *JAMA Netw. Open*, vol. 5, no. 6, 2022, doi: 10.1001/jamanetworkopen.2022.15276.

[3] B. J. Auerbach, S. Dibey, P. Vallila-Buchman, M. Kratz, and J. Krieger, “Review of 100% fruit juice and chronic health conditions: Implications for sugar-sweetened beverage policy,” *Adv. Nutr.*, vol. 9, no. 2, pp. 78–85, 2018, doi: 10.1093/advances/nmx006.

[4] L. E. Barry *et al.*, “An umbrella review of the effectiveness of fiscal and pricing policies on food and non-alcoholic beverages to improve health,” *Obes. Rev.*, vol. 24, no. 7, 2023, doi: 10.1111/obr.13570.

[5] M. A. C. Escobar, J. L. Veerman, S. M. Tollman, M. Y. Bertram, and K. J. Hofman, “Evidence that a tax on sugar sweetened beverages reduces the obesity rate: A meta-analysis,” *BMC Public Health*, vol. 13, no. 1, Nov. 2013, doi: 10.1186/1471-2458-13-1072.

[6] L. Hyseni *et al.*, “The effects of policy actions to improve population dietary patterns and prevent diet-related non-communicable diseases: Scoping review,” *Eur. J. Clin. Nutr.*, vol. 71, no. 6, pp. 694–711, 2017, doi: 10.1038/ejcn.2016.234.

[7] A. Itria, S. S. Borges, A. E. M. Rinaldi, L. B. Nucci, and C. C. Enes, “Taxing sugar-sweetened beverages as a policy to reduce overweight and obesity in countries of different income classifications: A systematic review,” *Public Health Nutr.*, vol. 24, no. 16, pp. 5550–5560, 2021, doi: 10.1017/S1368980021002901.

[8] S. S. Nakhimovsky, A. B. Feigl, C. Avila, G. O’Sullivan, E. MacGregor-Skinner, and M. Spranca, “Taxes on sugar-sweetened beverages to reduce overweight and obesity in middle-income countries: A systematic review,” *PLoS One*, vol. 11, no. 9, pp. 1–22, 2016, doi: 10.1371/journal.pone.0163358.

[9] H. Park and S. Yu, “Policy review: Implication of tax on sugar-sweetened beverages for reducing obesity and improving heart health,” *Heal. Policy Technol.*, vol. 8, no. 1, pp. 92–95, 2019, doi: 10.1016/j.hlpt.2018.12.002.

[10] E. Pineda *et al.*, “Review: Effectiveness and policy implications of health taxes on foods high in fat, salt, and sugar,” *Food Policy*, vol. 123, no. October 2022, p. 102599, 2024, doi: 10.1016/j.foodpol.2024.102599.

[11] L. M. Powell, S. Marinello, J. Leider, and T. Andreyeva, “A Review and meta-analysis of the impact of local U.S. sugar-sweetened beverage taxes on demand,” *Policy, Pract. Prev. Res. Cent.*, no. 121, p. Research Brief No. 121, 2021, [Online]. Available: https://p3rc.uic.edu/.

[12] M. Redondo, I. Hernández-Aguado, and B. Lumbreras, “The impact of the tax on sweetened beverages: A systematic review,” *Am. J. Clin. Nutr.*, vol. 108, no. 3, pp. 548–563, 2018, doi: 10.1093/ajcn/nqy135.

[13] M. Sassano *et al.*, “National taxation on sugar-sweetened beverages and its association with overweight, obesity, and diabetes,” *Am. J. Clin. Nutr.*, vol. 119, no. 4, pp. 990–1006, 2024, doi: 10.1016/j.ajcnut.2023.12.013.

[14] S. Sobhani and M. Babashahi, “Taxation for reducing purchase and consumption of sugar-sweetened beverages: A systematic review,” *Int. Arch. Heal. Sci.*, vol. 6, no. 2, p. 65, 2019, doi: 10.4103/iahs.iahs_62_18.

[15] A. M. Teng, A. C. Jones, A. Mizdrak, L. Signal, M. Genç, and N. Wilson, “Impact of sugar-sweetened beverage taxes on purchases and dietary intake: Systematic review and meta-analysis,” *Obes. Rev.*, vol. 20, no. 9, pp. 1187–1204, 2019, doi: 10.1111/obr.12868.

[16] K. Thiboonboon, R. D. A. Lourenco, P. Cronin, T. Khoo, and S. Goodall, “Economic Evaluations of Obesity-Targeted Sugar-Sweetened Beverage (SSB) Taxes–A Review to Identify Methodological Issues,” *Health Policy (New. York).*, vol. 144, no. April, p. 105076, 2024, doi: 10.1016/j.healthpol.2024.105076.

[17] A. Wright, K. E. Smith, and M. Hellowell, “Policy lessons from health taxes: A systematic review of empirical studies,” *BMC Public Health*, vol. 17, no. 1, pp. 1–15, 2017, doi: 10.1186/s12889-017-4497-z.

[18] D. Kim and I. Kawachi, “Food Taxation and Pricing Strategies to ‘Thin Out’ the Obesity Epidemic,” *Am. J. Prev. Med.*, vol. 30, no. 5, pp. 430–437, 2006, doi: 10.1016/j.amepre.2005.12.007.

[19] L. M. Powell, J. Chriqui, and F. J. Chaloupka, “Associations between State-level Soda Taxes and Adolescent Body Mass Index,” *J. Adolesc. Heal.*, vol. 45, no. 3 SUPPL., pp. S57–S63, 2009, doi: 10.1016/j.jadohealth.2009.03.003.

[20] K. J. Duffey, P. Gordon-Larsen, J. M. Shikany, D. Guilkey, D. R. Jacobs, and B. M. Popkin, “Food price and diet and health outcomes: 20 years of the CARDIA study,” *Arch. Intern. Med.*, vol. 170, no. 5, pp. 420–426, 2010, doi: 10.1001/archinternmed.2009.545.

[21] J. M. Fletcher, D. E. Frisvold, and N. Tefft, “The effects of soft drink taxes on child and adolescent consumption and weight outcomes,” *J. Public Econ.*, vol. 94, no. 11–12, pp. 967–974, 2010, doi: 10.1016/j.jpubeco.2010.09.005.

[22] J. M. Fletcher, D. Frisvold, and N. Tefft, “Can Soft frink Taxes Reduce Population Weight?,” *Contemp. Econ. Policy*, vol. 28, no. 1, pp. 23–35, 2010, doi: 10.1111/j.1465-7287.2009.00182.x.

[23] R. Sturm, L. M. Powell, J. F. Chriqui, and F. J. Chaloupka, “Soda taxes, soft drink consumption, and children’s body mass index,” *Health Aff.*, vol. 29, no. 5, pp. 1052–1058, 2010, doi: 10.1377/hlthaff.2009.0061.

[24] B. H. Lin, T. A. Smith, J. Y. Lee, and K. D. Hall, “Measuring weight outcomes for obesity intervention strategies: The case of a sugar-sweetened beverage tax,” *Econ. Hum. Biol.*, vol. 9, no. 4, pp. 329–341, 2011, doi: 10.1016/j.ehb.2011.08.007.

[25] A. D. M. Briggs, O. T. Mytton, D. Madden, D. O. Shea, M. Rayner, and P. Scarborough, “The potential impact on obesity of a 10 % tax on sugar-sweetened beverages in Ireland , an effect assessment modelling study,” *BMC Public Health*, vol. 13, no. 1, p. 1, 2013, doi: 10.1186/1471-2458-13-860.

[26] A. D. M. Briggs, O. T. Mytton, A. Kehlbacher, R. Tiffin, M. Rayner, and P. Scarborough, “Overall and income specific effect on prevalence of overweight and obesity of 20% sugar sweetened drink tax in UK: Econometric and comparative risk assessment modelling study,” *BMJ*, vol. 347, no. October, pp. 1–17, 2013, doi: 10.1136/bmj.f6189.

[27] J. M. Fletcher, D. E. Frisvold, and N. Tefft, “NON-LINEAR EFFECTS OF SODA TAXES ON CONSUMPTION AND WEIGHT OUTCOMES,” *Natl. Bur.*, vol. 1131, no. 2007, pp. 1127–1131, 2014, doi: 10.1002/hec.

[28] M. Manyema *et al.*, “The potential impact of a 20% tax on sugar-sweetened beverages on obesity in South African adults: A mathematical model,” *PLoS One*, vol. 9, no. 8, 2014, doi: 10.1371/journal.pone.0105287.

[29] S. Basu, S. Vellakkal, S. Agrawal, D. Stuckler, B. Popkin, and S. Ebrahim, “Averting Obesity and Type 2 Diabetes in India through Sugar-Sweetened Beverage Taxation: An Economic-Epidemiologic Modeling Study,” *PLoS Med.*, vol. 11, no. 1, Jan. 2014, doi: 10.1371/journal.pmed.1001582.

[30] A. H. Kristensen *et al.*, “Reducing childhood obesity through U.S. Federal policy: A microsimulation analysis,” *Am. J. Prev. Med.*, vol. 47, no. 5, pp. 604–612, 2014, doi: 10.1016/j.amepre.2014.07.011.

[31] M. W. Long *et al.*, “Cost Effectiveness of a Sugar-Sweetened Beverage Excise Tax in the U.S.,” *Am. J. Prev. Med.*, vol. 49, no. 1, pp. 112–123, 2015, doi: 10.1016/j.amepre.2015.03.004.

[32] J. L. Veerman, G. Sacks, N. Antonopoulos, and J. Martin, “The impact of a tax on sugar-sweetened beverages on health and health care costs: A modelling study,” *PLoS One*, vol. 11, no. 4, Apr. 2016, doi: 10.1371/journal.pone.0151460.

[33] A. D. M. Briggs *et al.*, “Health impact assessment of the UK soft drinks industry levy: a comparative risk assessment modelling study,” *Lancet Public Heal.*, vol. 2, no. 1, pp. e15–e22, 2017, doi: 10.1016/S2468-2667(16)30037-8.

[34] T. Barrientos-Gutierrez *et al.*, “Expected population weight and diabetes impact of the 1-peso-per-litre tax to sugar sweetened beverages in Mexico,” *PLoS One*, vol. 13, no. 1, p. e0191383, 2018, doi: 10.17605/OSF.IO/KHKWP.

[35] F. Schwendicke and M. Stolpe, “Taxing sugar-sweetened beverages: Impact on overweight and obesity in Germany,” *BMC Public Health*, vol. 17, no. 1, pp. 14–18, 2017, doi: 10.1186/s12889-016-3938-4.

[36] E. J. Bourke and J. L. Veerman, “The potential impact of taxing sugar drinks on health inequality in Indonesia,” pp. 1–8, 2018, doi: 10.1136/bmjgh-2018-000923.

[37] A. Saxena, A. D. Koon, L. Lagrada-Rombaua, I. Angeles-Agdeppa, B. Johns, and M. Capanzana, “Modelling the impact of a tax on sweetened beverages in the Philippines: An extended cost–effectiveness analysis,” *Bull. World Health Organ.*, vol. 97, no. 2, pp. 97–107, 2019, doi: 10.2471/BLT.18.219980.

[38] A. Saxena, N. Stacey, P. D. R. Puech, C. Mudara, K. Hofman, and S. Verguet, “The distributional impact of taxing sugar-sweetened beverages: Findings from an extended cost-effectiveness analysis in South Africa,” *BMJ Glob. Heal.*, vol. 4, no. 4, 2019, doi: 10.1136/bmjgh-2018-001317.

[39] P. M. Sowa, E. Keller, N. Stormon, R. Lalloo, and P. J. Ford, “The impact of a sugar-sweetened beverages tax on oral health and costs of dental care in Australia,” *Eur. J. Public Health*, vol. 29, no. 1, pp. 173–177, 2019, doi: 10.1093/eurpub/cky087.

[40] P. Wilde *et al.*, “Cost-effectiveness of a US national sugar-sweetened beverage tax with a multistakeholder approach: Who pays and who benefits,” *Am. J. Public Health*, vol. 109, no. 2, pp. 276–284, 2019, doi: 10.2105/AJPH.2018.304803.

[41] K. E. Kao, A. C. Jones, A. Ohinmaa, and M. Paulden, “The health and financial impacts of a sugary drink tax across different income groups in Canada,” *Econ. Hum. Biol.*, vol. 38, p. 100869, 2020, doi: 10.1016/j.ehb.2020.100869.

[42] N. Urwannachotima, P. Hanvoravongchai, J. P. Ansah, P. Prasertsom, and V. R. Y. Koh, “Impact of sugar-sweetened beverage tax on dental caries: A simulation analysis,” *BMC Oral Health*, vol. 20, no. 1, pp. 1–12, 2020, doi: 10.1186/s12903-020-1061-5.

[43] N. T. Rogers *et al.*, “Changes in soft drinks purchased by British households associated with the UK soft drinks industry levy: a controlled interrupted time series analysis,” *BMJ Open*, vol. 13, no. 12, pp. 1–13, 2023, doi: 10.1136/bmjopen-2023-077059.

[44] P. Phonsuk, V. Vongmongkol, S. Ponguttha, R. Suphanchaimat, N. Rojroongwasinkul, and B. A. Swinburn, “Impacts of a sugar sweetened beverage tax on body mass index and obesity in Thailand: A modelling study,” *PLoS One*, vol. 16, no. 4 April, pp. 1–15, 2021, doi: 10.1371/journal.pone.0250841.

[45] L. B. Nucci, A. E. M. Rinaldi, A. F. Ramos, A. Itria, and C. C. Enes, “Impact of a reduction in sugar-sweetened beverage consumption on the burden of type 2 diabetes in Brazil: A modeling study,” *Diabetes Res. Clin. Pract.*, vol. 192, no. September, p. 110087, 2022, doi: 10.1016/j.diabres.2022.110087.

[46] S. Liu, P. J. Veugelers, K. Maximova, and A. Ohinmaa, “Modelling the health and economic impact of sugary sweetened beverage tax in Canada,” *PLoS One*, vol. 17, no. 11 November, pp. 1–16, 2022, doi: 10.1371/journal.pone.0277306.
